# Supplementary material for: Insights into early animal evolution from the genome of the xenacoelomorph worm Xenoturbella bocki
Source: eLife. 2024 Aug 7;13:e94948. doi: 10.7554/eLife.94948 (PMC11521371; doi:10.7554/eLife.94948)
Supplement: Figure 8—source data 1. [file elife-94948-fig8-data1.docx]

**Neuropeptide Ligand Sequences**

**Novelty:**

Same as … > Sequence already published (>xxxx = published sequence)

New Xenoturbella sequence > other family member(s) already identified in Xenoturbella

New to Xenoturbella > other family member(s) already identified in Acoels and/or Nemertodermatida

New to Xenacoelomorpha > No other members identified in Xenacoelomorpha

**Sequence features:**

Signal peptide predicted with SignalP-5.0 (Almagro Armenteros et al. 2019)

Basic cleavage sequences and sites (known motif) and potential post translational modification sites (G, amidation ; E/Q, pyroglutamination) predicted with NeuroPred (Southey et al. 2006)

For sequences already published, *italicised amino acids* correspond to divergences between the already published sequence and the one reported here

**7B2 ____________________________________________________________**

>g13221.t1_Xenoturbella_bocki_7B2 (This study – New to Xenacoelomorpha)

MEPMAMVTRLLLILAGAVICQSYPYQQQQDTGTSLFKKMIDELINTRVEYPETNELPFDETPMKVEGGTHEGGQHLGPYGTFANEHEVLTDPYDLGYYSYYPPGEDSYESAIATNDEPPTEEDAAEKPYPAPVNPCPIGYSETDGCLEEVPDAAWFSKSYQESQVGKSDPEHNYDENPYRGGEKRLELVAKKAPIIRRRKRSAGPNPYLNGVKTRTVAKKSPTIR

**Bursicon alpha ___________________________________________________**

>g4833.t1_Xenoturbella_bocki_Bursicon alpha (This study – New to Xenoturbella)

MLTVLLVSALLIPTPCTTCNLRRAALRFSHPLCLTKVISTQMCAGYCQSYARVSPLGHASLDRTCRCCQPMMERKMSVELRCPLLKHRRMSIDIPVAIECMCRITSCGGDDYYGPSYDDASLYDASSSTHRHSPMLLWYR

**Bursicon beta ____________________________________________________**

>39242.1_Xenoturbella_bocki_Bursicon beta (Thiel et al. 2018)

MLTLLLLTLAVGWGAATEGTAETCHVVFSDTTIRQQVDYGAEGQAVCTGTVTLHRCEGNCYSQARPSVLHGFAS*SCNCCRETVLVETEVVLLDCFDGAGDGDHLPDVRYTLRIQEPVECACSRCYN*

>g10294.t1_Xenoturbella_bocki_Bursicon beta (This study – same as 39242.1 – Thiel et al. 2018)

MLTLLLLTLAVGWGAATEGTAETCHVVFSDTTIRQQVDYGAEGQAVCTGTVTLHRCEGNCYSQARPSVLHGFAS*VCIDQAWVTGGPRANLAPGLLKSGP*

**Glycoproteine Hormone alpha _______________________________________**

>26735.1_Xenoturbella_bocki_Glycoproteine Hormone alpha (Thiel et al. 2018)

MHHRRTLVIVVTLVVVVHCICPILTFPTLTNGLSPPMDDHTRLGGPLYFRGFHNGSRAVGEEVDSRILNVKIRENKARSKRSGIVGCHLVGYIQRVEIDGCTPVNVAMNACRGYCVSYAYPTNPGGPYLFTASTQCCRITERHRVPFIVECDNGGKYQGFFLSARACACGICDYES

>g13431.t1_Xenoturbella_bocki_Glycoproteine Hormone alpha (This study – same as 26735.1 – Thiel et al. 2018)

MHHRRTLVIVVTLVVVVHCICPILTFPTLTNGLSPPMDDHTRLGGPLYFRGFHNGSRAVGEEVDSRILNVKIRENKARSKRSGIVGCHLVGYIQRVEIDGCTPVNVAMNACRGYCVSYAYPTNPGGPYLFTASTQCCRITERHRVPFIVECDNGGKYQGFFLSARACACGICDYES

**Glycoproteine Hormone beta ________________________________________**

>4807.1_Xenoturbella_bocki_Glycoproteine Hormone beta (Thiel et al. 2018)

MKSTFAFLILIVCSFAAEGLATTTELDDLVMCVKREYRQHIASMSGCRDERILTVACWGRCETQMVPKLEPPYKESFHSVCIPYNYTIGQIQMQDCDEGVDPTYSFPQPGICMCQSCADQGYVVACH

>g13430.t1_Xenoturbella_bocki_Glycoproteine Hormone beta (This study – same as 4807.1 – Thiel et al. 2018)

MKSTFAFLILIVCSFAAEGLATTTELDDLVMCVKREYRQHIASMSGCRDERILTVACWGRCETQMVPKLEPPYKESFHSVCIPYNYTIGQIQMQDCDEGVDPTYSFPQPGICMCQSCADQGYVVACH

**Calcitonin _______________________________________________________**

>4670.1_Xenoturbella_bocki_Calcitonin (Thiel et al. 2018)

*MNNKTLYIVLS*MVLSTMLVLASSAPTDANSRKKRDLESDLAAVKFIEALLNLEDNVERISYEKELYEQPMPINMESNPVRRTDCSMSVCLQSQIAHALLSRPKGPDTGANSPGK

>g8753.t1_Xenoturbella_bocki_Calcitonin (This study – same as 4670.1 – Thiel et al. 2018)

MVLSTMLVLASSAPTDANSRKKRDLESDLAAVKFIEALLNLEDNVERISYEKELYEQPMPINMESNPVRRTDCSMSVCLQSQIAHALLSRPKGPDTGANSPGK

**GnRH-AKH ______________________________________________________**

>6444.1_Xenoturbella_bocki_GnRH-AKH (Thiel et al. 2018)

*MFTIYSQA*MPRMDRRAMMAIGLVLMLVVQSCLAANGFTGSSNWLPAGKRSFMDQETPAPDDVEENGGEKTIGPCVCAVLDNGRRYFKTVINYMLWDEKQAHNDRMQANRRDLLDDKLWL

>g6494.t1_Xenoturbella_bocki_GnRH-AKH (This study – same as 6444.1 – Thiel et al. 2018)

MPRMDRRAMMAIGLVLMLVVQSCLAANGFTGSSNWLPAGKRSFMDQETPAPDDVEENGGEKTIGPCVCAVLDNGRRYFKTVINYMLWDEKQAHNDRMQANRRDLLDDKLWL

**Insulin-like peptide a ______________________________________________**

>20871.1_Xenoturbella_bocki_Insuline-like peptide a (Thiel et al. 2018)

*MGIVTMVCMSLLVLLAVIGGSDAVNRHLCGAELANTLRMLCGDRGYNAPQYEGAHGVMSHSHYTIPVFRTKRAAHNYLGAVVPNR*MKRGTGRIVQECCRQTCSLSNLALYCAPERLPIDISSENSEESFEFLETSVDTTSAESATDGVEEGEYSSGDSELNEVEVIDNDGTNMIAYR

>g7805.t1_Xenoturbella_bocki_Insuline-like peptide a (This study – same as 20871.1 – Thiel et al. 2018)

MKRGTGRIVQECCRQTCSLSNLALYCAPERLPIDISSENSEESFEFLETSVDTTSAESATDGVEEGEYSSGDSELNEVEVIDNDGTNMIAYR*RRRRDIEQLSAEYGFGDNLDNADTSRRELWRKWQGLPVRRQTSLDFTDDDEDTVGNSPGWIRFGLPR*

**Insulin-like peptide b ______________________________________________**

>g7804.t1_Xenoturbella_bocki_Insuline-like peptide b (This study – New Xenoturbella sequence)

MMLKSYATIRYNVCVLFLVLALVSQQQYRADAAFLCGSQFPRALRNACSTLTKRSLENALFRDQADNAIQNDKRFDYMADYCCYHGCQMSQLVFFCRK

**Insulin-like peptide c ______________________________________________**

>g7806.t1_Xenoturbella_bocki_Insuline-like peptide c (This study – New Xenoturbella sequence)

MVNTTCLCVYIALFLTPVVLSMNDLVEDAEISKRREWHCNNGVAETLHMLCSGCYAGTIGKRYSDIDEFMLSEKVARSFLGRTIVPAVGKRGVIDECCLRRCAVPEMMGYCC

**Insulin-like peptide d ______________________________________________**

>g7807.t1_Xenoturbella_bocki_Insuline-like peptide d (This study – New Xenoturbella sequence)

MALPGFNVSACKTGGVYAWLVVAVLVISAVHGQSQWHCRDSVPALMQAICGGCYRIHNSQSSAHTAEDYIIQADDYNSPRVEIEEQYNAQDSAFLDKKRALNYLTPDVFKRQVTIGIIDECCRRQCSFSELSAYCGVPGIDC

**Prokineticin 1 ____________________________________________________**

>rna_35664.1_Xenoturbella_bocki_Prokineticin 1 (Thiel et al. 2018)

FGGVFTREDRFPKTIQRCSEDVDCPVSHCCAYSLFAQLKECKPLGSEGDTCNVFSFPYAYDGDRQGRLCPCRRHLLCN

>g2577.t1_Xenoturbella_bocki_Prokineticin 1 (This study – same as rna_35664.1 – Thiel et al. 2018)

*MMDRTGSLVFLIILSVFVHCARGNGL*FGGVFTREDRFPKTIQRCSEDVDCPVSHCCAYSLFAQLKECKPLGSEGDTCNVFSFPYAYDGDRQGRLCPCRRHLLCN*RPIHEGLGVCQTDLSSSKKLRPSSYW*

**Prokineticin 2 ____________________________________________________**

**(Not found in the genome presented here)**

>rna_22210.1_Xenoturbella_bocki_Prokineticin 2 (Thiel et al. 2018)

MLGERCVGFRGIVLTTCVVYTVVVLVTVTSAYHGLSLNTADEPPFSEDSLYTNQGIVLDQVRKRSKTSSRTRHEVYNVFRRAPPVEVCYKDADCRPHGCCVRSHYIPTINQCRPLAGAGQKCAPPDLFIRGLRDTDYCPCTASVTCVKVNRKDSFGYCLA

**Vasotocin _______________________________________________________**

>7489.1_Xenoturbella_bocki_Vasotocin (Thiel et al. 2018)

MYRTVFIYTLVTVLSLYADVASSCLVQGCPIGGKRSMNDAERQCSACGPGYRGVCVGLQTCCGDFGCHMGTDDAKMCLTEQINPEPCHVEGRKCGLNAYAKCVADGICCDFETCTLDEKCQQIGEGHDSWPANNNDAGVGRITAFLRSLRADQ

>g6592.t1_Xenoturbella_bocki_Vasotocin (This study – same as 7489.1 – Thiel et al. 2018)

MYRTVFIYTLVTVLSLYADVASSCLVQGCPIGGKRSMNDAERQCSACGPGYRGVCVGLQTCCGDFGCHMGTDDAKMCLTEQINPEPCHVEGRKCGLNAYAKCVADGICCDFETCTLDEKCQQIGEGHDSWPANNNDAGVGRITAFLRSLRADQ

**NucB2/Nesfatin ___________________________________________________**

>g3978.t1_Xenoturbella_bocki_ NucB2 (This study – New to Xenacoelomorpha)

MGVMLVINAVLLMVMVTDMSAAPLSPTFNETQPQNDTDGKGLDALEYERYLRQVIEVLETDKNFKEKINNADIDDIRTGKIANELKFVKHNVRTKLDELKRREIDRMRTVLKEKYALERGLSPIEAEEFKEMISHMDHENPDKFETSDLEKLIKKATYDLEQMDSQRRDDFKKYEMDKEVKRRQKMNDLSDEERVAEKQAYDSMQAKHKDHEKVKHPGSKAQLEEVWEETDGLDKEDFDPKTFFKLHDTSDDGYLDLSEVMALFQKELEKVYEDSNEEDDMMEFDEEMNRMREHVMTEVDLDKDGLISLEEFLQYAQNSDFDKDDGWETLDEQQLYTEDELKSFEDELRKEEEALRQKKAELEKLRQQQEALRQRTASRPDPQRTEPPPPLEVEVPIDGGEGQSETDELIYKP

**LRFDIamide _____________________________________________________**

>836.1_Xenoturbella_bocki_LRFDIamide (Thiel et al. 2018)

MKLFDLFCVTLVAGIASVYCDAEEGFMPASSDVDKRLKFDIGKKRHFDDKRLRFDIGRKRAWEEGQENDYAQELVLGMADGVHDYLANNADDSVSKRLYDMSKRLRFDIGKRLKFDIGKRLGADESNDLVVIGGVEIPVCAQEDEPGLSLCGFAPMGGRWWPICSDNCEELKADSYD

>g4607.t1_Xenoturbella_bocki_LRFDIamide (This study – same as 836.1 – Thiel et al. 2018)

MKLFDLFCVTLVAGIASVYCDAEEGFMPASSDVDKRLKFDIGKKRHFDDKRLRFDIGRKRAWEEGQENDYAQELVLGMADGVHDYLANNADDSVSKRLYDMSKRLRFDIGKRLKFDIGKRLGADESNDLVVIGGVEIPVCAQEDEPGLSLCGFAPMGGRWWPICSDNCEELKADSYD

**SFxNamide ______________________________________________________**

>12867.1_Xenoturbella_bocki_SFWNamide (Thiel et al. 2018)

MNYYIYPLFLAILLWYQLPLTASEETLADYMKEDGTTDSGIGIRSFWNGKRAWADQGLDEMINEEARAFWNGKRSFWNGKRSFWNGKRAPVETDFDEDKRSFWNGKREPDVGENYDDALLKKSFWNGKRSFWNGKRSFWNGKRADDSQREDIPPVEYMELFDRLFGHQSDGKLAP

>g6941.t1_Xenoturbella_bocki_SFWNamide (This study – same as 12867.1 – Thiel et al. 2018)

MNYYIYPLFLAILLWYQLPLTASEETLADYMKEDGTTDSGIGIRSFWNGKRAWADQGLDEMINEEARAFWNGKRSFWNGKRSFWNGKRAPVETDFDEDKRSFWNGKREPDVGENYDDALLKKSFWNGKRSFWNGKRSFWNGKRADDSQREDIPPVEYMELFDRLFGHQSDGKLAP

**PxFVamide ____________________________________________________**

**(Not found in the genome presented here)**

>21216.1_Xenoturbella_bocki_PxLFVamide (Thiel et al. 2018)

MTNMAIISVCVLLVLAVNIVNGSADFCEQFPDLCDDAEMSKRQLNVFPWYEVWNSGKRQDVEIRREPPLFVGKRREPPLFVGKREEASYFVGEKK

**LxFamide _____________________________________________________**

**(Not found in the genome presented here)**

>1521.1_Xenoturbella_bocki_SLQFamide (Thiel et al. 2018)

GRRSLQFGRRSLQFGRRSLQFGRRSLQFGRRSLQFGRRSLQFRHLSMSHARRFPNTQQGRR

**Achatin _____________________________________________________**

>2558.1_Xenoturbella_bocki_GFGN peptide (Thiel et al. 2018)

MSCTSVTVCYWLLMCVLMCATVLSTPVGELGVYDDADLKDNLGDISDTSAQETARLVSSCLSYVSELMRSDVDNALMLDID*DRGFGNKRIAGFGNKRIPGFGNKREPGFGNKRGFGN*

>g14892.t1_Xenoturbella_bocki_GFGN peptide (This study – same as 2558.1 – Thiel et al. 2018)

MSCTSVTVCYWLLMCVLMCATVLSTPVGELGVYDDADLKDNLGDISDTSAQETARLVSSCLSYVSELMRSDVDNALMLDID*RLLSVT*

**LRIGamide____________________________________________________**

>g2539.t2_Xenoturbella_bocki_LRIGamide isoform a (This study – New to Xenoturbella)

MTCKLSCVLLVAFLFFVCTLAAPYFDEAEIEELYNEAKALEAEEEGDFDEAKRLRIGSKRGAMAVRLGGKRFEFEDEDMQDEDKRYALRIGSRDPDPLRIGSRDPEALRIGSRDPLRIGSRDPNPLRIGSRDPNPLRIGSRDPEPLRIGSRDPKPLRIGSRDPEPLRIGSRDAEPEALRIGSKRSAIAVRVGGKRDPLRIGSRDPLRIGS

>g2539.t1_Xenoturbella_bocki_LRIGamide isoform b (This study – New to Xenoturbella)

MTCKLSCVLLVAFLFFVCTLAAPYFDEAEIEELYNEAKALEAEEEGDFDEAKRLRIGSKRGAMAVRLGGKRFEFEDEDMQDEDKRYALRIGSRDPDPLRIGSRDPEALRIGSRDPLRIGSKRSAIAVRVGGKRDPLRIGSRDPLRIGS

**AVW peptide _____________________________________________________**

>g1644.t1_Xenoturbella_bocki_AVW peptide partial (This study – New to Xenacoelomorpha)

HQGQHRGAGRCLEEGAAVWKRGQLSGRGDSCLEEGTAVWKRGQLSGRGDSCLEEGAAVWKRGQLSGRGAAVWKRGQLSGRGDSCLEEGTAVWKRGQLSGRGDSCLEEGAAVWKRGQLSGRGDSCLEEGTAVWNSMC

**FWxVW peptide __________________________________________________**

>g6256.t1_Xenoturbella_bocki_FWxVW peptide (This study – New to Xenacoelomorpha)

MKLSTNSVIALVLCLSVFVQTSHALWRVWKQDPLIGKRSFWQVWKQNEQADAGKRAFWRVWQDSGENSKRFAKSWQDDTRYDGEEGMNTADSLGENMGGAYDLPLEDYITEFKEMDTNDDGIVEMNEYLVSRGATGDIELEE

**APVAPQExTGQ/LTRSG peptide ______________________________________**

>g11181.t1_Xenoturbella_bocki_APVAPQExTGQ/LTRSG peptide partial (This study – New to Xenacoelomorpha)

TRASVTANQSSKRASNAPVAPQEETGQAVSSSSRENESDGDDRRLTRSGTNQRSTRASVAANRPSKRASPAGTAPAPVAPQEDTGQAVSSSSRENESDDRRLTRSGTNQRSTRASVAANRPSKRASPAGTAPVAPQEDTGQDSDADDRRLTRSGATARSSTASTRAGAKRPATHKRI

**pyroWVP peptide _________________________________________________**

>g8585.t1_Xenoturbella_bocki_pyroWVP peptide partial (This study – New to Xenacoelomorpha)

QRVGTPSSGYSSEWVPRRQGTPASGYPVVRVLQRVGTPSSGYSSEWVPRRQGTPASGYPVVRVLQRVGTPSSGYSSEWVPRRQGTPASEYPVVRVLQRVGTPSSGYSSEWVPRRQGTPASGYPVVRVL

**TTE peptide ______________________________________________________**

>g3343.t1_Xenoturbella_bocki_TTE peptide partial (This study – New to Xenacoelomorpha)

HGGIDVGRDVAAGVMAGGNIGAATVNNFYYGTGTDSGRTERKRRSENDGVKTTERKRRSENDGVKTTERKRRSENDGVKTTE

**Other potential candidates __________________________________________**

>g617.t1_Xenoturbella_bocki_candidate1 (This study)

IRRHEDRRGEEKRKREVEKRSEEKRRSEEKKERREEKRRSEEKKKEEKRREEAKVN

>g14917.t1_Xenoturbella_bocki_candidate2-a (This study)

VLDINPRKVLDINPRKVLDINPRKVLDINPRKVLDINPRKVLDINPRKVLDINPRKVLDINPRKVLDFNH

>g440.t1_Xenoturbella_bocki_candidate2-b (This study)

VFLEDYSVLSSQAIRGEVAAGLSKEMLGVELKADETYAITAKLGKISIKEVDKKKLAGQLNNGYCYGQRRSQGASLRKVLDINPRKVLDINPRKVLDINPRKVLDINPRKVLDINPRK

>g3274.t1_Xenoturbella_bocki_candidate3 (This study)

MSDVTDAITLEDSDDDDVVLDEWTLVDRQGDECLLPTANRRAPPDSHIPAEIKGSTFDDDDAGHRRSETSGSESPDIAADDIIVLDCQGCDGNFVREEERSDPLLCEGERSDPLLSEEERSDPLLSEGERSDPLLSEEGRSDPLLSEGEREAECSVTELVQEGCSVLHTCILSCDEQELRAEVAHSFHVDAEQAQKAQLLSTY

>g6769.t1_Xenoturbella_bocki_candidate4 (This study)

MARTPGRVRPMACTPGRVRPMACTLGRVRPMVCTPGRVRPMACTPGRVRPMACTLGRVRPMTGKPGRVRPMTGKPGCVRPMACTLGRVRPMTGKPGSLRSMTGKPGRVRPMACTLGRVLPMACTLGRVWPMACTLGRVRPMACTLGRVRPMACTLGRVRPMTGKPGRVRPMTGKPGSVQPMACTLGRVRPMTGKPGRVRPMTGKPGSVRPMTGKPGRVRPMACTLGRVRPMACKLGRVRPMTGKPGRVRPMTGKPGRVRPMTGKPGSLRSMTGKPGSLRSMTGKPGRVRPMACTLGRVRPMACTLGRVRPMACTPGRVRPVIGKPGRMGSMAPGSYNVCDFLVSSQADPTPESHSGPLLRRNILAKPLWIARDSPEFRDCQIELTRPFRVSSICPGSSLKRAAH

References:

Almagro Armenteros, J.J., Tsirigos, K.D., Sønderby, C.K. *et al.* SignalP 5.0 improves signal peptide predictions using deep neural networks. *Nat Biotechnol* **37,** 420–423 (2019).

Bruce R. Southey, Andinet Amare, Tyler A. Zimmerman, Sandra L. Rodriguez-Zas, Jonathan V. Sweedler, NeuroPred: a tool to predict cleavage sites in neuropeptide precursors and provide the masses of the resulting peptides, Nucleic Acids Research **34 suppl_2**, 267–272 (2006).

Daniel Thiel, Mirita Franz-Wachtel, Felipe Aguilera, Andreas Hejnol, Xenacoelomorph Neuropeptidomes Reveal a Major Expansion of Neuropeptide Systems during Early Bilaterian Evolution, Molecular Biology and Evolution **35-10**, 2528–2543 (2018).
