# Supplementary material for: Insights into early animal evolution from the genome of the xenacoelomorph worm Xenoturbella bocki
Source: eLife. 2024 Aug 7;13:e94948. doi: 10.7554/eLife.94948 (PMC11521371; doi:10.7554/eLife.94948)
Supplement: Figure 8—source data 2. [file elife-94948-fig8-data2.docx]

**Neuropeptide Receptor Sequences**

**Novelty:**

Same as … > Sequence already published (>xxxx = published sequence)

New Xenoturbella sequence > other family member(s) already identified in Xenoturbella

New to Xenoturbella > other family member(s) already identified in Acoels and/or Nemertodermatida

New to Xenacoelomorpha > No other members identified in Xenacoelomorpha

Potential xxx Rc > Sequences branching at the base of the group to which they may belong

**Sequence features:**

For sequences already published, underlined amino acids correspond to divergences between the already published sequence and the one reported here

**Rc types :**

**Secretin GPCR / Rhodopsin GPCR / RTK / LGR**

**Calcitonin/DH31 GPCR**

>Xenoturbella|X.boc|9189.1|Calcitonin/DH31_GPCR_homolog_1 (Thiel et al. 2018)

MSVSTEGFESSTMSSASISPTTGVNYTDSGGGPTEILSTRTYQKLYEECMAKLDNLPTPDYPVYCPNHFDEWGCWEDTPNGTVAIISCPQIDGFDTSKFAYKECMPNGTWWTHPETQHMWADYTNCHLDEEWGEEMVAVSKFITVGYAISFIAVIGALFIFIYFKALHCQRNTIHLHLLMTFAMSCTAHIIWFPWMIHMRETAVYPEACKFVYVLTLYFQCSTYFWMLCEGLYLHTLIVVAVFSVKKNLLLYYLLGWGTPCVLILIYVILRLTIHAVDETIACWVTVDVISYVIVMPECGTLVVNVVFLLNIVRVLVTKLRASNSPEQNQYRKAVRATLILIPLLGMWMFLFFIDPPDESMYVLRMIYKYINGFCLSFQGSFVACIYCYFNGEVIATIKTFYLRQSMIRSLSTKPQLTREQCTSNSPNKILSSSSELKGESPV*

>Xenoturbella|X.boc|g6990.t1|Calcitonin/DH31_GPCR_homolog_1 (This study – same as 9189.1 – Thiel et al. 2018)

MSSASISPTTGVNYTDSGGGPTEILSTRTYQKLYEECMAKLDNLPTPDYPVYCPNHFDEWGCWEDTPNGTVAIISCPQIDGFDTSKFAYKECMPNGTWWTHPETQHMWADYTNCHLDEEWGEEMVAVSKFITVGYAISFIAVIGALFIFIYFKALHCQRNTIHLHLLMTFAMSCTAHIIWFPWMIHMRETAVYPEACKFVYVLTLYFQCSTYFWMLCEGLYLHTLIVVAVFSVKKNLLLYYLLGWGTPCVLILIYVILRLTIHAVDETIACWVTVDVISYVIVMPECGTLVVR

>Xenoturbella|X.boc|17452.1|Calcitonin/DH31_GPCR_homolog_2 (Thiel et al. 2018)

MATDDSTADPNIVAVICGINVTNTSSIFTDGGRYCPAMFDTWACWNYTAAGNVSYISCPPLPGFDTSHFAYKECYPNGTWYLSSEERPLGFGNYSGCYAEYPGFSPLIYLMLAGSGISLVTVILAIIIFAYFKALHCQRNTIHMHLLVTFAMSRIAFILPFLGWNMEGVMCKIMMVLNEYCECANYFWMLCEGVYLHTLIVVAVFTEQKSLRWFYVLGYGVPALPTLLYVILEATVGKANENSGVQDCMTGDKLLYATKIPIVIALLLNAVFLLNIVRVLVTKLRASQSPEQNQYRKAVRATLILIPLFGLWVFLMFVEPKGNKTASSIYQYTNGFLQSFQGFLVACIYCFFNGEVKTQFRRKWERRQLNRSKMDRKGSRSFSLYTTTDYL*

>Xenoturbella|X.boc|g14660.t3|Calcitonin/DH31_GPCR_homolog_2 (This study – same as 17452.1 – Thiel et al. 2018)

MATDDSTADPNIVAVICGINVTNTSSIFTDGGRYCPAMFDTWACWNYTAAGNVSYISCPPLPGFDTSHSTSSQYTRGLNFAYKECYPNGTWYLSSEERPLGFGNYSGCYAEYPGFSPLIYLMLAGSGISLVTVILAIIIFAYFKALHCQRNTIHMHLLVTFAMSRIAFILPFLGWNMEGVMCKIMMVLNEYCECANYFWMLCEGVYLHTLIVVAVFTEQKSLRWFYVLGYGVPALPTLLYVILEATVGKANENSGVQDCMTGDKLLYATKIPIVIALLLNAVFLLNIVRVLVTKLRASQSPEQNQYRKAVRATLILIPLFGLWVFLMFVEPKGNKTASSIYQYTNGFLQSFQGFLVACIYCFFNGEVKTQFRRKWERRQLNRSKMDRKGSRSFSLYTTTDRHLPVVNGNGHHDTTL

**CRF/DH44 GPCR**

>Xenoturbella|X.boc|9963.1|CRF/DH44_GPCR_homolog (Thiel et al. 2018)

MNITDTTAASNFTAMTSLEPTTAVPLLLTTTPETSNVTESMYKVFAQPMIPASLYERIHDKAIIAGYNLCYTRYRNFTYLATDIYCPPLYDGVLCWGAVPGDTNSTIACPEQLHGVKYINGSTSTKACLSDGTWAKAIYNCIEPQPANDITHSQSLTYLTVYVIAIVISLIVCIGAFTIFTVFYSRIKCLRNIIHMNLIGTFILRNILQIMLFANVLVEQQSAESDSSSGSSIYCRALYTLFYYAVLSNFFWMFVEGLYLVSLVFFAMSSRKLGFWFYCIIGWAIPVVITLIWVTVVSQYRTESRCWTAKQGDDNYEFEYIIHAPIYTVMFINLVQMVLIVGMLFTKLKASSSLETAQYRKAVKAIVVLLPLLGLTYALASVSPTEDQALQLAFMYINALLQSTQGIMVSVVYCYLNSEVKTLLMKRFGYWKDTRNFHTTRTSRGPSLSLVETNQNHFQSVKYRIGQQNSENGVSTV*

>Xenoturbella|X.boc|g14639.t2|CRF/DH44_GPCR_homolog (This study – same as 9963.1 – Thiel et al. 2018)

MNITDTTAASNFTMTSLEPTTAVPLLLTTTPETSNVTESMYKVFAQPMIPASLYERIHDKAIIAGYNLCYTRYRNFTYLATDIYCPPLYDGVLCWGAVPGDTNSTIACPEQLHGVKYINGSTSTKACLSDGTWAKAIYNCIEPQPANDITHSQSLTYLTVYVIAIVISLIVCIGAFTIFTVFYSRIKCLRNIIHMNLIGTFILRNILQIMLFANVLVEQQSAESDSSSGSSIYCRALYTLFYYAVLSNFFWMFVEGLYLVSLVFFAMSSRKLGFWFYCIIGWAIPVVITLIWVTVVSQYRTESRCWTAKQGDDNYEFEYIIHAPIYTVMFINLVQMVLIVGMLFTKLKASSSLETAQYRKAVKAIVVLLPLLGLTYALASVSPTEDQALQLTFMYINALLQSTQGIMVSVVYCYLNSEVLEEFKKRLSVWRDMKSFTSLPLHRITGFLSWEFTLKAPLGMGRNVIEVTRIVKCQGVKP

**PDF_GPCR**

>Xenoturbella|X.boc|9675.1|PDF_GPCR_homolog_1 (Thiel et al. 2018)

MDPVLLAAIGSRNVSDLMSPANQKECRTTIEETEASMTLDVFCKGDWDVVCWLPIEPNTTQHQPCPDVFGFDASKEATRICGSDGLWTPGVGGRISDFYPCVSSDAQGVMDALGGNADEQTRFSEIASDARVMEIVGYCVSFVSLAVAMFIFCYFKCLHCSRIHIHKQLFLSFMIHALMNIIITGDQLRFLNDQEGFRSTPIVCETFEVLLQYCKLSAFSWMFVEGLYLHGLIAVSVFQGRPNFFLYYFIGWGTPVFILIPWVVVECLYNTDVCWLGYNLVSKNPYFWIIEVPRNIVLLVNLVFLLSIIRVLIVKLRATNTSETEQVRKAVKAAIVLLPLLGLTNILFIAPPPKASDPTSVIILGLWGSLFLQSFQGFFVALIYCFLNGEVRAALVKHWKRWQTWSSTGTRRRASRSLSMFTSTTEVPMKHLYAEGARVSPKL*

>Xenoturbella|X.boc|g14633.t1|PDF_GPCR_homolog_1 (This study – same as 9675.1 – Thiel et al. 2018)

MRRGRRRLGLGIFDDVFQTSRKSLSDEMLVAAIGSRNVSDLMSPANQKECRTTIEETEASMTLDVFCKGDWDVVCWLPIEPNTTQHQPCPDVFGFDASKEATRICGSDGLWTPGVGGRISDFYPCVSSDAQGVMDALGGNADEQTRFSEIASDARVMEIVGYCVSFVSLAVAMFIFCYFKCLHCSRIHIHKQLFLSFMIHALMNIIITGDQLRFLNDQEGFRSTPIVCETFEVLLQYCKLSAFSWMFVEGLYLHGLIAVSVFQGRPNFFLYYFIGWGTPVFILIPWVVVECLYNTDVCWLGYNLVSKNPYFWIIEVPRNIVLLVNLVFLLSIIRVLIVKLRATNTSETEQVRKAVKAAIVLLPLLGLTNILFIAPPPKASDPTSVIILGLWGSLFLQSFQGFFVALIYCFLNGEVRAALVKHWKRWQTWSSTGTRRRASRSLSMFTSTTEVYCFCFHHVHPTMP

>Xenoturbella|X.boc|g14668.t1|PDF_GPCR_homolog_2 (This study – New Xenoturbella sequence)

MFLTEFVDQARTVPEPPPRADIDSGTIEIEQNDGVGIVERIVRDALLTERSTTTETTETTDLFTTDVTFLNDMESGGTAFDNGTGDGQATNVTIHYLSLDELLATFMRQMELDCLRNFENTAYPDDEREKNLPKMELSDRAVETLSSSDSSSTQREVSRDCDRGASGTVGIAPEHPPHAAWITYYIYYVGCCLSLTCLTATLAIFCYFRNLACARISIHKHLVVSFILYNICLIVQSHRVDGTGWDDQNGYMWKGHMKLVWCRMLLWMVWFTRLANMSWMLVEGLYLHERIAVSVFNTEPKYKLYYFLGWGCPIIFTSACSAVMHYVSKDDCFQDASSLHWIWIVVGPRTLALLLNLSFVVNIVRILLTHQRPKSNDEGAQLRKAAKATVVLIPLLGVGNLIVLANPRDDGAGETIFMISNAIIQSSQGILIAVIYCFTSREVHTAIKREIHKYKRRKQPFSTTGRSRLSTSGLSRTNSLFVSENYPRPTSFV

**PTH_GPCR**

(Not found in the genome presented here)

>Xenoturbella|X.boc|15571.1|PTH_GPCR_homolog (Thiel et al. 2018)

MSVSPDVLMELGMNSSDLTTLQYMETFSLDVLSKILINEVRCRLDMMNSPTPDYDVYCPETWDGFICWPDTKADTNVQLPCPSFQHIDDTQHDPDQGRATRYCGRNGTWEAKGNETSITNMDNCEFDALGSIELPKFMVHGQLIFTIGYAFSLISLTISMIIMSRLKRLHCTRNIIHMHLFSSFILRALFYFAKEGTKKYGASNNMFGIVACRMVTVAWHYFILTNVFWILVEGLYLQTLLFSAVFSAKRWMRIYYILGWAVPASFPACLTLFRLLIENTECWLHYIKYPHATWFIRAPLIGVVITNFVLFLNILRILLTKLKASKSPQTRQLRYRYRIRCSIYAASDIGTQSVYMGPSASCCYQHPPFTP*

**Galanin Rc**

>Xenoturbella_X.boc_g8203.t1_Galanin_GPCR_homolog (This study – New to Xenacoelomorpha)

MAYRTGGRGRGALTGQGSVDGPNRVAMITPIFSLSRIGIPVNPKMGFYRAKKWTYRYRFRHLFIAMLVSIQFDHAMSGNPGSTSTTESTTVDTSAATDEESWMWTARWHTLDSRNPWYALYVSLLVLFSVVLVVGLAANVAVMAMIIRSGKYRLNTTNIFILNLNIADMLFLLICLPTHASVLSFEEWLFGETMCKISHFMMYASMVASILTMVVMSVDRMVAVHYPLRTITMRTTQRAVISCTLIWIIAVVVSGPTLYTYTTMSSDSVEYGYVRYYCFDPWEDNASGRLTYVICMFLISFVLPFIAVTFCYVKLVRSLWTRVAPTSNINQLGSKRKVTRMVMTVVLVFGVCWLPYHIMLIWRSTPTFPYTTLSYVFQTSAFILSYSNACLNPLLYAFLSDNFRRIFLKGCGSHSNRVDPILARGQMPLNNTPNQMRTTGRTNMPPNNPSIPSSSDLNGLPRLNASH

**AllatostatinA Rc**

>Xenoturbella_X.boc_ g8228.t1_Allatostatin_A_GPCR_homolog (This study – same as 13387.1 – Thiel et al. 2018)

MANTTFSPSIINTSDLTSIGNTTMGPPNSDDGVIISYVLGVIFVAIIIIGLIGNLLVTIVIIKDKQMKNTTNVYIFNLAMADLLVILFTVPISTYQNFNGNWIFGEFVCQITYYLQFVTLYISIYTLVLMSVDRYLAVVHAIKSMSYRTASNALISVVVIWVITITVMIPVPMSTHLVKLAPELMHCAFYPLFDEYGNIDQNRLNQNYNVFLYIFLITSYVIPLLVITVLYMVLLRRIWEVKPGGNMSAESQKSKRRVTRMVVAVITVFAVCCLPMQISFLLQAMNMWDRENVAMGVLNMLARVMVYSNSAVNPIIYAFMSDNFRQAFAKLFPCLGKMFLKRPQNNEKSKRYGYSVGRTETSAA

>1564_Xenacoelomorpha_Xenoturbella_bocki_13387.1_Allatostatin_A/Galanin_related_rhodopsin_type_neuropeptide_GPCR_complete (Thiel et al. 2018)

MANTTFSPSIINTSDLTSIGNTTMGPPNSDDGVIISYVLGVIFVAIIIIGLIGNLLVTIVIIKDKQMKNTTNVYIFNLAMADLLVILFTVPISTYQNFNGNWIFGEFVCQITYYLQFVTLYISIYTLVLMSVDRYLAVVHAIKSMSYRTASNALISVVVIWVITITVMIPVPMSTHLVKLAPELMHCAFYPLFDEYGNIDQNRLNQNYNVFLYIFLITSYVIPLLVITVLYMVLLRRIWEVKPGGNMSAESQKSKRRVTRMVVAVITVFAVCCLPMQISFLLQAMNMWDRENVAMGVLNMLARVMVYSNSAVNPIIYAFMSDNFRQAFAKLFPCLGKMFLKRPQNNEKSKRYGYSVGRTETSAA*

**Somatostatin/Allatostatin Rc**

>Xenoturbella_X.boc_g3545.t1_Somatostatin_MHC_Allatostatin_C_GPCR_homolog1 (This study – New to Xenoturbella)

MPVHGDDNFGSCASAQVEGMVMAMIDAGGDKDETVNHSIMTSLSVIGVKQAKTVLGCCSSYLKKHHNLPEQHRIIILNVMERIIKQMDIKLDAPFAVSIIKLASDEMTKTKDVMPDWQTAASGLMVAMGATHCHEAIEELRTKFVPGGLPHFFVIQTLANLSVASVYEVVPYLTEFIGMMLTMIGMVRHDNMKWVFSYALSKFSESIIDYTANLENAPDPTIRIEMYYTPVYSAFDVLFGQWLQSKESKLRLIIVEAVGQMAYLIAPSRIDEQLPRIISGFLALYRKHPEPFHITQGLCMVLDAAVKAGCTTLDAMFDNLLSCLFPQREGMTTVSPSAAESDLMTSVNWGGSELSGGNTTSLSLVVPEPMSVVIVACVVFCIVFVVAFVSNILVIYVVITFKKMRTIPNIFILNLAISDALLSCSLPVLAYNYVSDQWQLGAAFCKIHIVVDGIAQFSTVYSLTALTIDRYVAIVFPLGCRIKVNRIRTAVIACGCLWVLATVVSTPIFMFTGIQDYDGWIACDGNFPNALFFCVYMIVMAFLIPYALICYSYIMILADLWSNRRQQYLDGVTCNSNTSRKITKVVVCVCAGFLVCCTPTYAMVFVSAKYELTGEQPTIALTTVHKVALALNYIHSFLNPIIYACLSESFRRHFCLIMPCLRRISTQRLSNDDSPPSARLNTKQSTLL

>Xenoturbella_X.boc_g8453.t1_Somatostatin_MHC_Allatostatin_C_GPCR_homolog2 (This study – New to Xenoturbella)

MGFDNFTDDYNTTGASAYYVLVMGITLQFSAVILWIACILGLIGNSLVIFVILRNSKMQTVTNIYILNLAIADFVFMVVEILLGSDYYNLRVWSYGIEMCRIMLYVDRLNQFVSIYLLMVMSVDRYLAVVHAIKSSQFRTCTIAAITSAMVWLISMFIAIPAAVNSMLVGQRPDHMACHSDPKIWELWVNIIACYFIPLVVIVTCYALMIIRLRSVKPKGLKPERRKKSTRKVTRLVISVVAAFIICWTPFYLVLTVRLCIQMYSLPLEILGTLTIPLAAFNSCLNPFLYAFLSDNFRKAFFRVFRCTSAFDRSMSHSGAAGARSQGTNANTRVEAAKRNAARTDTVNACDANNAFKMKRLRSDSVNVNGPMTSLTTCSPKSSSDEDK

**KISS peptide Rc**

>Xenoturbella_X.boc_g6850.t1_Kisspeptin_GPCR_homolog (This study – New to Xenacoelomorpha)

MDLNLTSTDVSTNITHSEQPSYEIIVPVFFGIVTFFGILGNAIVIFVIAMHTHMRSVTNYYIVNLALGDLVFLVVCAPLTAAALAQPSWTFGEILCKAIMYIMEVTAIATCITLTAMTIDRFLVIIYPMESLRGRRPSKAIAISCGVWMFSLLCPVPLAIFMEVVEIGGATHCIEVWPTETWKVVYYVYIFIVAYLLPMVVITVCYIAMLRVLWSGMVPTGDSQQSAKSLSKRRRVTFMILIVVVLFAGCWLPFWVVTLMEKIQPQIARVQGAQVAKLVVLCLAYGNSCLNPFVYAFMGENFQQRLKETCSMNFWRGGRAALRRSTIRTRTQTATVSLRDVNSKQINGVGV

**Tachykinin GPCR like Homolog**

>Xenoturbella_X.boc_ g1096.t1_Tachykinin_GPCRlike_homolog partial (This study – same as 15475.1 N-term – Thiel et al. 2018)

MDNVSDFPVSTAPSLFPSTDLGLSTNGPSSTPLSDFDYAQGTVAAMFWAVAFAFLTITAAGGNLVVVWIVLAHKRMRTVTNYFLVNLAISDACIAIFNVTFNVTTFILYDWVFGDVWCVLIRYMGPLFVVVSVLTLAALSADRYRAIVMPMRPRLSRSTTKLIIAAIWVISLVMCSPYAIYAETHVLSNLPDGSSTRMCWLNWPGGPYSQTELKFLTPI

>Xenoturbella_X.boc_ g1097.t2_Tachykinin_GPCRlike_homolog partial (This study – same as 15475.1 C-term – Thiel et al. 2018)

MVTYCIPLTVLAVTYSIIGVNLWGNITPGETTNRYQEQLNSKRKVVKMMIVVVIVFATCWLPLHIYKVLEDVNGYLFQAKYLTHVYMALFWLAMSHSMYNPFIYCYMNDRFRRGFRKCFQCFPCVKWSRDDTIDGQKSRFASTYAHRLERNGATTATGIYDDEDHRI

>1566_Xenacoelomorpha_Xenoturbella_bocki_15475.1_Tachykinin_related_rhodopsin_type_neuropeptide_GPCR_complete (Thiel et al. 2018)

MDNVSDFPVSTAPSLFPSTDLGLSTNGPSSTPLSDFDYAQGTVAAMFWAVAFAFLTITAAGGNLVVVWIVLAHKRMRTVTNYFLVNLAISDACIAIFNVTFNVTTFILYDWVFGDVWCVLIRYMGPLFVVVSVLTLAALSADRYRAIVMPMRPRLSRSTTKLIIAAIWVISLVMCSPYAIYAETHVLSNLPDGSSTRMCWLNWPGGPYSQTELKFIILQVMVTYCIPLTVLAVTYSIIGVNLWGNITPGETTNRYQEQLNSKRKVVKMMIVVVIVFATCWLPLHIYKVLEDVNGYLFQAKYLTHVYMALFWLAMSHSMYNPFIYCYMNDRFRRGFRKCFQCFPCVKWSRDDTIDGQKSRFASTYAHRLERNGATTATGIYDDEDHRI*

>Xenoturbella_X.boc_g1095.t1_Tachykinin_GPCRlike_homolog2 (This study – New Xenoturbella sequence)

MYDYRIFFQTPTMADNTSAMTPTPPVSSTMPPNIDFSLSTIAILLWSIPYLFLVVTAIVGNLVVVWIVLAHKRMRTVTNYFLVNLAISDIFIAIFNVTFNAITFMIGDYIYGSAMCRTLSMMGPLFVIVSVLTLSAISADRFRAIVMPMMPRMSKMVTKMVIVSIWTVAVLMCLPIAVFSEMFVIPLDNGTDSYNICVTMFFGDQYATSDLVFRVTQMLVTYVVPLVVMTIAYSIIGVNLWGNVIPGETSNRYQEQLVAKRKIVKMMVTVVVVFAICWMPFHITRVLENLNGYLFAGKFVVHLYLAIFWFAMSHSIFRRGFRKCFRCCPFVKWAKNDTIDAPKVKYVSTYASRVNRNGSTMTTATACDDDDDDHHL

>Xenoturbella_X.boc_g12868.t1_Tachykinin_GPCRlike_distant_homolog (This study – New Xenoturbella sequence)

MATLTTLNLTDSVNATNTAMTSDDTDDAVYPLWRLAVWCVIYGIVIVLAFGGNVIVIWIVTTQKSMRTMTNLFLVNMAFSDIMVASLNMPWYFSVNVNDSGEWPYGGDSTFGIAFCRTVRFVGHVSTTASIFSMTAIAIDRYMAIMSPMRYKITKVKAIAGIIVIWVLTALICSPDIAYGMVYYREHSRIYVCAFSDAAAFIIYKWMDFIFTYLVPLAMITVVYAIVSKELWMQQTIGECSNAKQLKSKRKVVKMMVTVVVLFALSTVPSHILRIMFAHNVKFDGDVLKELNLSVTLGINLNSAYNPLIYMFRVGFVRFFVTIAGIRKMPQTVGNQYDSRSRRSKANTVVSEFPLETRTSAKTRGSSSDA

**RYamide / luqin GPCR like homolog**

>Xenoturbella_X.boc_g1522.t1_RYamide_Luqin_GPCRlike_homolog_1 (This study – same as 15306.1 – alternative start – Thiel et al. 2018)

MSSPDPTLFDPANESGVGSFNGSTTTETQTDPDYNGSWVPSFIEPKSIQAAWGLCYALITVLAIGGNFIVCYIIVSTKRMRTVTNYFIFNLALSDFLLAVICINPTFYPALLSYWPFGAIMCPCVMFFQSASVLVSSYTAVAICLDRYVAIIYPLTPRMTRTCAILVIVLIWAIAFTISTPIAVVTRLSAPYRSYSDDIVNDCVENWPSEKSNIMYSQSLLVLQYFLPLMMLLFGYLRISFSLWGQKVPGEGESIRDAKQTMAKKKLIKMLIVVVVIFGVCWLPLNVFILITANDKTLYDYMHINHIYFACHWLSMSSACVNPFIYCYMNARFRNGFIFFFHQVLPCLPTKRDQSALNGSTRHTFVSNLSSFRSRSSRGSSLRRSPATHTKISKV

>1565_Xenacoelomorpha_Xenoturbella_bocki_15306.1_Luqin_related_rhodopsin_type_neuropeptide_GPCR_complete (Thiel et al. 2018)

MEMSSPDPTLFDPANESGVGSFNGSTTTETQTDPDYNGSWVPSFIEPKSIQAAWGLCYALITVLAIGGNFIVCYIIVSTKRMRTVTNYFIFNLALSDFLLAVICINPTFYPALLSYWPFGAIMCPCVMFFQSASVLVSSYTAVAICLDRYVAIIYPLTPRMTRTCAILVIVLIWAIAFTISTPIAVVTRLSAPYRSYSDDIVNDCVENWPSEKSNIMYSQSLLVLQYFLPLMMLLFGYLRISFSLWGQKVPGEGESIRDAKQTMAKKKLIKMLIVVVVIFGVCWLPLNVFILITANDKTLYDYMHINHIYFACHWLSMSSACVNPFIYCYMNARFRNGFIFFFHQVLPCLPTKRDQSALNGSTRHTFVSNLSSFRSRSSRGSSLRRSPATHTKISKV*

>Xenoturbella_X.boc_g1519.t1_RYamide_Luqin_GPCRlike_homolog_2 (This study – insertion with repeats in C-ter - New Xenoturbella sequence)

MSGLFGPFNSSHDPVWASTTVPGDVNNSLAQSTPFQNDDTLFDKPLYVKLIWGVMYAMITLLAIGGNVLVCVIVLSNHRMRNVTNYFLVSQAVSDILMAVFCIIPTYYSTQQNEWVFGAVLCPVVLYTMSITVTVSVFTMIALALDRYVAVLHPLKPRMSHRAGKMSIAGIWAAAAFIGLPVALFSRKTVKFGGEGSDCYEVWPRGQSEYHYSVGVMCIQYFVPLVVLCVAYGRICCKLLGREVPGERDNARDAKQAAATKKLIKMLIIVVIMFAICWLPLYTFTIAQSAHGEILYYPHIDIIYFACHWISMTSAMWNPIIYFSLNRNFRRGLKKLLHCREMNNYPEFTSQRNSSMLMREIRKSEKRCSRQRPFHELTAEGLAGLGYKGLAGLGYKGLAGLGYKGLAGLGYKGLAGLGYKGLAGLGYKGLAGLGYKGLAGLGYKGLAGLGYKGLAGLGYKGLAGLGYKGLAGLGYKGLAGLGYKGLAGLGYKGLAGLGYKGLAGLGYKGLAGLGYKGLAGLGYKGLAGLGYKGLAGLGYKGLAGLGYKGLAGLGYKGLAGLGYKGLAGLGYKGLAGLGYKGLAGLGYKGLAGLGYKGLAGLGYKGLAGLGYKGLAGLGYKGLAGLGYKGLAGLGYKGLAGLGYKGLAGLGYKGLAGLGYKGLAGLGYKGLAGLGYKGLAGLGYKGLAGLGYKGLAGLGYKGLAGLGYKGLAGLGYKGLAGLGYKGLAGLGYKGLAGLGYKGLAGLGYKGLAGLGYKGLAGLGYKGLAGLGYKGLAGLGYKGLAGLGYKGLAGLGYKGLAGLGYKGLAGLGYKGLAGLGYKGLAGLGYKGLAGLGYKGLAGLGYKGLAGLGYKGLAGLGYKGLAGLGYKGLAGLGYKGLAGLGYKGLAGLGYKGLAGLGYKGLAGLGYKGLAGLGYKGLAGLGYKGLAGLGYKGLAGLGYKGLAGLGYKGLAGLGYKGLAGLGYKGLAGLGYKGLAGLGYKGLAGLGYKGLAGLGYKGLAGLGYKGLAGLGYKGLAGLGYKGLAGLGYKGLXQLSLPSLPHRPTQPSIPPSQTNSAFHPSLTDQLSLPSLPHRPTQPSITPSQTNSAFHPSLTDQLSLPSLPHRPTQPSIPPSQTNSAFHSSRVGE

>Xenoturbella_X.boc_g1098.t1_RYamide_Luqin_GPCRlike_homolog_3 (This study – New Xenoturbella sequence)

METTGPTPGAVLTTMLPDLASFFTTDLIDDVNLTVATIDENREIFNEPLSVKITWGALYFILSLVALGGNCIVCYIILSSARMRNVTNYFIANMAISDAMMAVICVNPAYWGPVLRYWPFGEFLCRVGFFVQSVSVGVSVLSIAAICVDRYVAIIYPLRPRMTHRVALLCTGAMWALSSAVALPTLLNAKYVKYESDDGGVFHDCVEEWTYKFYYSIALMIWQYFVPLFVLSVGYGRIIWTLWYQRIPGEREEMRDAKQAASKRKLIKMLSVVLLTFALTWLPMQVFTLSVHANKLLLKYKHINHIYFASHITAMASTACNPIVYYYMNGRFRNGFRRVFAKCLPCCSIPAPDEMSLGRTNTNVTRIDSTCYQMVTPGNGKRTQARSPTAGSPTNYRKVNGVSQI

**tFMRFa GPCR like homolog**

>Xenoturbella_X.boc_g11066.t1_tFMRFamide_GPCR_homolog (This study – New Xenoturbella sequence)

IHDPWEYELLPKYAVVLAVVLYGLVSVVSLLGNIVVVVVLTVGTRTPSKLNVFLINLAISDILMACFCVPFTFTGFTFGRWPFSDGLCPVVMAIQSVSVTSSVYTMMAVGLDRFYAVFWPLKSRMTRGRFVVAKIIVALIWLLSFGAGAAPLVSSGVTWYLVLLDGDPMPYHLPRCEENLGWTSGTRLAYNMTFFALCYAGPLIVLAFTYGSVAYCLWSRTTPGNADETRDIAQEHAKRRVIKMLVVIVAAFAFCWLPLNAFNIIRDFNFSLLLQMDDMTHMGVFFACHWLAMSHSVANPLIYGFLNQNFRVGNQRVLA

>Xenoturbella_X.boc_g1115.t1_tFMRFamide_GPCR_distant_homolog_1 (This study – same as 34982.1 – Thiel et al. 2018)

MSASIAITIIRHMYNNAAFAINGRGIMSSFSYAYGAEMSDLDGTPDVTEPGSMDFEGNMTLFNDSQMCRNTESYLTVPVQIAVIIAYSFASLASIFGNVMVVFVLAYGPRAKADVNTFLINLAVCDILMGIFCIPFSFVDGIYLRWIFGPIMCPLVKFVQVVSVSGSVFTLSALGWDRYCAVYRPIQSHTRHSRFKLSLVFVWIAAIAIGSVQLIVTRMQKCSICVETYSTDRELNRTLRITYTVVVFFATYFIPLCIMMFTYLSIAHKLWGHRTPGNANETRDQQQTRSKKKVTLMVMIIVVLFAICWLPTHVFSQMETFKQGQFFDRNDSYVMMTLVFLGVNWLAMANSFVNPIIYSFLNENFKADLKTVFGFMKSRNASRKENRKISTSSYTYLRPTSLRTMRRTGTVKTADGDDAAAGINDGDTAVDGLQWDELRASTTGRSLHSGAPGTTMRSGAI

>1573_Xenacoelomorpha_Xenoturbella_bocki_34982.1_trochozoan_FMRFamide_related_rhodopsin_type_neuropeptide_GPCR_internal (Thiel et al. 2018)

DSQMCRNTESYLTVPVQIAVIIAYSFASLASIFGNVMVVFVLAYGPRAKADVNTFLINLAVCDILMGIFCIPFSFVDGIYLRWIFGPIMCPLVKFVQVVSVSGSVFTLSALGWDRYCAVYRPIQSHTRHSRFKLSLVFVWIAAIAIGSVQLIVTRMQKCSICVETYSTDRELNRTLRITYTVVVFFATYFIPLCIMMFTYLSIAHKLWGHRTPGNANETRDQQQTRSKKKVTLMVMIIVVLFAICWLP

>Xenoturbella_X.boc_g1000.t1_tFMRFamide_GPCR_distant_homolog_2 (This study – New Xenoturbella sequence)

MAGSTVASTVDDFINGTTINSTWSYPSLEMTLTQQIPLIILYAITSILSVTGNVFVIIVLSCGTRSRSDVNIFLINLALCDILTGCLVMPFTFVEVMLIRFIFGYIMCPLVKFIELVSVSGSILTLTALGIDRFQAVMRPLQSHLSRSRAKISIISVWVVSLALGGVQLFSTRLDAQSYCQERPDLKIIYTYLVFGALYIAPLAILIVTYAMIGKRLWGHQTPGNADYARDKSQAKSKKKVVLMPMVLVTLQRTYSAQVPMSQTGM

>Xenoturbella_X.boc_g1126.t1_tFMRFamide_GPCR_distant_homolog_3 (This study – New Xenoturbella sequence)

MAELLLTNFSLNSTDLDGCDAKTPPDKYAFPLHTQIPLIILYSLTSVTSVIGNAIVILVLSCSSKGRSDVNTFLINLAVCDILMGLFATPFSFTEVMYVRWVFSPVMCPIVKFFTVTSVTSNVFTLTTLGIDRFQAVMWPMQRHMAKSRATIMLAFVWGFSFPLGAVQFFVRIVVCQTCLEDSRVFDRDSYTLAVFIATFVIPLAVLAFSYTCIGVKLWGRQTPGNADEARDKTHAKNKKRVVKMLATVVILFAVCWLPLHIFTFISDSLDKNTQMSHYRTLQWIYVSVHWLSNANSFVNPIIYSYFNETFRNDLKRLCSCIWGGRDEAQMTGRRFSARSTRLSSLYESFRHRNHPRNSHASTHAYELTINGSKAATREYSPSRNRLILSSDI

>Xenoturbella_X.boc_g1127.t1_tFMRFamide_GPCR_distant_homolog_4 (This study – New Xenoturbella sequence)

MALWQNGTFPLPSNATDGNFTMMDMCAMKAPMEDEFALAVSVQIPLIIIYSVTSLAIVGGNCLVIVVLTCNGKAHSDVNTFLINLAVCDILMGIFVTPFSFTEVMYVRWMFSAVMCPIVKFFAVTSVTGSVFTLTTLGIDRFQAVMWPLQRHMAKSRATIMLAFVWACALSLGAVQFFVSIVVCNNCLEDSRVFDRDSYTLAVFIATFVIPLAVLAFSYTCIGVKLWGRQTPGNADEARDRTHAKNKKKVGIRVSYTRAYCPCPMPGYGIIGGSSNSLGFGPHFSNTDQGP

**Leucokinin Rc**

>Xenoturbella_X.boc_ g5505.t1_Leucokinin_GPCR_homolog (This study – same as 20779.1 – Thiel et al. 2018)

MGSLLARQMCPGNYVGDDANVTVFSANETYNANETNYNPAMFDEPPTIKIILTIICVIICTLAVVGNFIVICVVVRSKELRTVTYALVANLSIGDIIIGMLSIPFSFYHQLMQRWDFGPVMCTTASFIQCVSVYISVYTLMAIALDRYFAVVHPLKQYMTARRAFIIVILVWVVGVVASFPGAYYTTTVTVPHTQRIVCWRGWPCTRMANGYTMFLVITQYIVPLGVIAGCYIIICHKLWSARKPGAASSEKDTKQAKNKKMQTQNGPALRRITNTVTVFQPINFSVKFSCTAQL

>1570_Xenacoelomorpha_Xenoturbella_bocki_20779.1_Leucokinin_related_rhodopsin_type_neuropeptide_GPCR_complete (Thiel et al. 2018)

MGSLLARQMCPGNYVGDDANVTVFSANETYNANETNYNPAMFDEPPTIKIILTIICVIICTLAVVGNFIVICVVVRSKELRTVTYALVANLSIGDIIIGMLSIPFSFYHQLMQRWDFGPVMCTTASFIQCVSVYISVYTLMAIALDRYFAVVHPLKQYMTARRAFIIVILVWVVGVVASFPGAYYTTTVTVPHTQRIVCWRGWPCTRMANGYTMFLVITQYIVPLGVIAGCYIIICHKLWSARKPGAASSEKDTKQAKNKKMQVIRMLIVVLSMFACCWLPIQVYRVLSVVYPDVILYEHINITHILLYLTAMSNSCCNPFIYSYYYLKGRRRSSASRATREKSSGLTLRSFVSNVGGTTKA*

**Prokineticin Rc**

>Xenoturbella_X.boc_g8043.t1_Prokineticin_GPCR_homolog_1 (This study – New to Xenoturbella)

MDATTIFPLINVTLFGNNTSTTNLTYGSDYEYYYPEEHSVIMKVVLCIMYLSIILICGTGNSLLLAVLIRVNHLRSVTYVLIANLTTSDLIVAVFCVPFFLVNVVLDRHWPFGQFMCAALAYINTVSLYVSTNTLLVIAADRYIIVRHPLQPRMSRRTTIIVIVCIWLVAFIFASPMIFTNETGLHINNDFKVVTMCFENWPADYHIVRKVYKMWIMVVQYLLPTSVMCYCYWQICYSMWTRKVPGTQTGQQEEEVIETKKKVVRRLIVVTAAFILCWLPYYVYAVYIYTFHMYDFYAWAFYLEMFFVVEAISLLNGIIDTCVYVILNKRIRKEIMKMFQSPINKSGHPTVRSAVRDTTNTRMIPLSSFRSNDSHSYT

>Xenoturbella_X.boc_g5628.t1_Prokineticin_GPCR_homolog_2 (This study – New to Xenoturbella)

MVEYYEDIVSTYPPFSEVVCSRAATSLDNDTYAAILAELIDINGEMVLNLTRSSVLQLMKYHCADSGDSYDSERVAAVDISVKTILAIVYILMIVLCGVGNTILLIVLYQFNNLRTVTTIFIANLTTSDLVVAVFGAPFNLVSFMLDSRWIFGDAMCPLVYYLNSTSLYVSTNTLLVISLDRLMLVMYPVKPRMRRRNASILIVGIWLWSGLISIPVAFFTKTSATGTAVACLEEWPALAAEYRTAYTLALFGIEFCAPFTIMVCSYSVICYKLWTREIPGTQTTEQEEATMESKRRVIRKLLLVTAMFFICWMPYYVWNLRSVNDGGELQNWRYGIPLFYLAEALSLSNSIVDTLVYVVFNRNVRDKFFKKKQKFTDAAELRSLNAAGSAFRSDKVVT

>Xenoturbella_X.boc_g2879.t1_Prokineticin_GPCR_homolog_3 (This study – New to Xenoturbella)

MAVNSTALVSSFDPPSDPGNGVDFVTLLCTLNTLEPVVRDSIVDRFNRANATTPMEDFIALMCSGSSGEGDNYVETMQTVTKVVMSVVYVLMIVICGVGNILFLVVVVTSKQLRKMTDVMIANLLLSDLVVALLCAPLNLVTVLNNRSWVFGSFLCPVVTYMNATSLYVSTNTLLAIAIDRLIVVANPLSRRTGYRTISIAFTIVIMIWVISGGVAVPVAMVTENQALHPGGNISARFCLETWEGQMNRYTVIYKTMTFALEFVVPILIMCVCYSCISCRLWHRQVPGVQLSDQEDAISDSKKRCIRRLVIVTAFFFLLWAPYYAVTLWWCLQTSTERFLFLEYQVTFAYVVESLSMSNSIVDTVVYVFLNRRVMKEAKQLYHEWCRTTRLRKRTSVRQESTKMTRLSSIKTVGF

**GPR83**

>Xenoturbella_X.boc_g6780.t2_GPR83_GPCR_homolog (This study – New to Xenacoelomorpha)

MDDDMDVFMIPYLDEDVLMSFRTNSSWNFSIFDDLLNDISWNHDMYIEASGGMHPAVKGVFVLLYCAIIVVSLFGNLTVCHVVYKNRNMRTVTNMYLVNMSISNVLITAVNIPVRIVTPILHDQWVMGEALCHINASTVTLSVFVCSFTMTLIAFDRHRAVIFPLKRKYSVTVGGLIIAAIWVVALGLSLPNAVFTKLEVYEDHSWARCSKYYPAWMTADTSPDTCHLVHTIIDFVLQFVMPLTVICALYTRISLRLFRRKVTGDNFNNKNSNAKRNSQLRRKRLTVKMMMLVCVVFAVCWLPYSLVLLSRHVVSDAAGTSLHVLAMSSVCYNPFIYCWLNKGYRKGFLAFKTAGLDRFRRRNESVSSPHSQRLTTTAPPKTTDTTQM

**NPY/F Receptor**

>Xenoturbella_X.boc_g4729.t1_neuropeptide_Y_F_GPCR_homolog (This study – New to Xenoturbella)

MSSTTALGATSSTNGSDSYNMSNTTPLWTSAWEDVVWVHQTIWFITLTVSIHVALIAIGLFGNGLVIFVILKTKRLHTVVGMFLGNLAVSDILITVLCMPVMLHYGLTESWSFGKVMCHAVFGAQGISMYVSAFTLAAIAVDRYRVIVYPFKNRLRVRHCYVIIALIWATACFISLPLFIFRKFEEINFPYYNIHVISCHEQYLAIPVSPSDVAMFKTLYTLLVFAATFLVPLIITSVAYIKIWLVLSHADTALSSNTIDNPAEAHKAVQNKRKAVKMLALVVVCFAVLWLPNHLFYILSDLAPELVFSASAYDKVASMSCNMVAWSSACCNPFLYGWLNARFRREFKSVFRCFGLGGTSRSFTNPKTKITEICNPTERSSVSVVVAPNRSRRHSSNL

**CCAP Rc**

>Xenoturbella_X.boc_g4534.t1_Neuropeptide_S_CCAP_GPCR_homolog (This study – New to Xenoturbella)

MWNYILPELMTAIDNEPEYEITSQLLESLAKVMPTIASSAVLTAMCVDRYFAIVHPMNLNTGNRSKYMIIVAWCFSVAASIPTTLMFRLQVVGGATMCWAAPELLHSQLYIVFVSCVSFFFPFLSILYCYSLIIITVSKRSKMMVGVSKPAKKKSFTQVLSFKRNNASENSKSMKAVKFDMHNRASSRGLIPKAKLKTIRMTLVICLVFVLCWAAYWPYYIGIIFGMFAENNKVQSIVQNLSTLNSCCNPIIYALFSTRVCEELRCICQRKSTTSTNPQYRDRRFRACDWVARKVCCCVCSPKPPYAPPNGRPSTRYTIDTHMTAAASSSSEERINGVRKGNQHMKHLNHTTACTSI

**Vasotocin Rc**

>Xenoturbella_X.boc_g4517.t1_Vasotocin_GPCR_homolog (This study – same as 19174.1 – Thiel et al. 2018)

MAFNFTTPYPINGTLPNTTFPDNATDATPFGRIESLAMFEHATNGVIFGVTVISNGCALLALWQMKKRLTRMLKFITHLCIADIMVAIFSVMYLLIYKLTFLFYGGWFMCSLMSYLQLFVTYLSTNVLMMMAVDRYMAICHPMATYLTYMTVTTRVYIMIGTAYLVSALFAIPQLFIFGLLEVGPNQYLCHSVAFMEPEHGAYYGLIYVTWVGVSVFFVPLLVLIFCYGEICRLVWFKHARLIAKQKQDTCTKGKGLLRGHNSNTDNKIIPRSHSVKRFSRAKVKTVKMNLTIIFAYIICWAPFWTAQFYSVYSEVGRKYYHTSPANTIVMLLSSLNSCVNPWIYMVFSGNMMGELKKLITCTNWHIGALFNFNGFSETGGDVSASTSADTVFSTCLLTTPTISRTPSPGFNQEPQQSSSIV

>1567_Xenacoelomorpha_Xenoturbella_bocki_19174.1_Vasotocin_related_rhodopsin_type_neuropeptide_GPCR_5prime_partial (Thiel et al. 2018)

GLLEVGPNQYLCHSVAFMEPEHGAYYGLIYVTWVGVSVFFVPLLVLIFCYGEICRLVWFKHARLIAKQKQDTCTKGKGLLRGHNSNTDNKIIPRSHSVKRFSRAKVKTVKMNLTIIFAYIICWAPFWTAQFYSVYSEVGRKYYHTSPANTIVMLLSSLNSCVNPWIYMVFSGNMMGELKKLITCTNWHIGALFNFNGFSETGGDVSASTSADTVFSTCLLTTPTISRTPSPGFNQEPQQSSSIV*

> Xenoturbella_X.boc_g4563.t1_Potential_Vasotocin_GPCR_homolog (This study – Potentially new Xenoturbella sequence)

MFNNSSSGLVQRNEHLARVEIGVTAAQVALGFLGNSCVLLGLYRRRELTRMHVFMMILCVGDLMSTFFTMLPQLIWKITEEFYAGEFICHLVTYLQCVAVYVSAYSVVALSIDRMSAICDPMRIHTSTNSRIILMVVLVWILALVFSIPQLFIFQLATVNGKAQCWPIYTEEWTRKAYITFLTSAVYVVPIAFIVCCYVRIIRTLQKNSKSFSEFRRSQKSEDQKKGSPTVDPSFAASTVRISRAKIKTVKMCAVIVTAYIVCWTPFFFVTMLTVWDKKSAALMFSTEPTVPVAIMAITMLMYSLNACVDPWIYLAFSGHLLHDVVRCLSCGLVEMNKASKSSKSTIKRSQQSQGSHMTQSRNAGRNAKKQDYSSLPQHKALDKTELETDL

**Achatin Rc**

>Xenoturbella_X.boc_g3997.t1_Achatin_GPCR_homolog_1 (This study – New to Xenoturbella)

MEGPTLEEMEGPTLEETVSFDSDNVSSARGNSTADEIWSTEVIVHVTVILIIMVVNLVGNLFIIFVLGCSKLARTFSSSVHMFILQLSIADLLVCLFTMNVEILFVAFGEWVLDTAACKISVYFQIVTLAASTFLLAAMSIDRFCTVCRPFDAPMTKHRARLMVVAAWVAALLLAIPQIFIFVVTERVGLGGRVVHQCVSGGYSSEWQRKAYFTWMMFYILVIPSVIIASCYLNIVLAVRGQMKQMHHAAPRESVDQMTSLSRAKRKTLVLSSCIIVTFIVCWSPYFVVTLAQIYGADVSKMVLIVIETLAMSNSAVNPWIYSCFYLHVRRIVCEYCCLVFPRPRCPNPIQSLQMRRDTTKTSSLSRRPRERIQMMHL

>Xenoturbella_X.boc_g3989.t1_Achatin_GPCR_homolog_2 (This study – New to Xenoturbella)

MIIIAWLMAFLFASPQILIFIQVSSKNDAGVVRKECLSQGYTAEWQRKMYFTFMTTYILIIPSIVITFCYFTIVRTMCQQQKEDHFSDLGSSSNGQCAGKKGGAAGGKRSIAFMLRSGRGKERPATTMSVRRGVSRRNISKAKSKTIKMSLCIIITFIVCWSPYFIVTMYYDIYGRHGAPPQMYVFIETMALINSATNPIWYGMFNLKLKSSLMAVFFPGKYAEHKRTLSTSRSYRITDTDNVSASDMGYRYSMKQRLNQRTGGGGTQSPKTTPRNNNTNGSSVEMLHYGRATPVSSPRTNSRLTVAEPHAIPTTETPLIAQ

>Xenoturbella_X.boc_g3992.t1_Achatin_GPCR_homolog_3 (This study – New to Xenoturbella)

MLNNSSLFDDGLYERRNGGSSLESHTIQRIVTLAGLILVALVGNTTVLCVLTCNNVARRRNRRVNIFVLNLAIGDLVVCFITMPTEIVWYVRDDWILGSFGCKFVAYIQVATIASTIFLLIVMSYDRYQAIAKPLHFSNAIRNAKVMVVCAWMLAAILAIPQIFIFNTTTVEDNGLFYLKCKSDGYTSDWQRRVYFGWFTTYILVIPTCLISFCYIQIIITVWRHGRSSGDAPQLRCTGSANISRAKVKTIKMTLCIIISFVMCWAPYFIVTNIEVFSSFRRIEPIWAFSETLALANSAVNPVLYSCFNLKLQNLMCRIFCPATWLLRKKSKSSRYTSKKTQSTSLAMSIHNNSTRTFSRNHRNKLATYPNSSVASTTVNVTTVIGPSEPSPVLTHHKNTVARLHGANDTNCNESNL

**GnRH/AKH Rc**

>Xenoturbella_X.boc_ g6043.t1_GnRH_AKH_GPCR_homolog_1 (This study – same as 20313.1 – Thiel et al. 2018)

MDQLNSTNTTMAIPTVYIFSNTTNTTTYPVQWHDLKAVYGPGYMLRATILVLIFIISLLGNTFLAVTLIKNKRRRSRMHALILHLAAVDLLFTFFVILQDFIWDITFIWAASNAMCKLSMFLKIYCLFISVFILVVITIDRWSAILFPLSSSNANVRCKVMLVVAHLLAIAMASPNLFIYSKQTHPHIPEFIQCVSLGNISDLSQWVFNVSVCLLQYIIPLGIMIYCYSAILHTIMKASRTSEDTGECRLRRTGTNSFPRAKVKTVKMTACLLATYLIVWTPYQVVQLWMIFEKGVGEKHPELTNALQIFGLTNAATDSIVYGFWNINFKKELARLCPRVAMKLFGPEVFADAAGNNRTTMSRMSVSRTCSTAHPTPTARNNGYSEAQRPNIANRPQNVEFNMKSFN

>1568_Xenacoelomorpha_Xenoturbella_bocki_20313.1_GnRH/AKH_related_rhodopsin_type_neuropeptide_GPCR_complete (Thiel et al. 2018)

MDQLNSTNTTMAIPTVYIFSNTTNTTTYPVQWHDLKAVYGPGYMLRATILVLIFIISLLGNTFLAVTLIKNKRRRSRMHALILHLAAVDLLFTFFVILQDFIWDITFIWAASNAMCKLSMFLKIYCLFISVFILVVITIDRWSAILFPLSSSNANVRCKVMLVVAHLLAIAMASPNLFIYSKQTHPHIPEFIQCVSLGNISDLSQWVFNVSVCLLQYIIPLGIMIYCYSAILHTIMKASRTSEDTGEGRLRRTGTNSFPRAKVKTVKMTACLLATYLIVWTPYQVVQLWMIFEKGVGEKHPELTNALQIFGLTNAATDSIVYGFWNINFKKELARLCPRVAMKLFGPEVFADAAGNNRTTMSRMSVSRTCSTAHPTPTARNNGYSEAQRPNIANRPQNVEFNMKSFN*

>Xenoturbella_X.boc_g2754.t1_GnRH_AKH_GPCR_homolog (This study – New Xenoturbella sequence)

MGASGRREPLGNGSAVNDTEPTWVYPGELSSYDSTLKIAGLSTIMVLAIIGNSTLLITMLRNRKKRSRINLLITNLALVDLFIALVMIPANISWHLSIGWYVGDFLCKLFMYTRLVSSLMSAFVLVVISLDRLLAILYPLSNVYAFTWCKIMLGVAYGISGLLSIPQLVFYWAGSPFPGFTQCTTMRRLTGSAVIAYNIYTPQVLYTIPLVMIIVSYSCILGYLIRTGRKKAFQDQGGLRRSGESAIPKAKIKTLKMSVCIILAFIVCQTPYHIENMYHVFTGNDTFLSTFISELFSLLQYSNIAIDPIVYGFFGINFQKELRRCCGKMDNKRSRGAGNNRSVTSMVCLSESASTRATINRQYKPSIRTDSTP

**Corazonin Rc**

>Xenoturbella_X.boc_g7935.t1_Corazonin_GPCR_homolog_1 (This study – New to Xenacoelomorpha)

MVNYSTSTPEYSWDASDGTDQYILYFSAQFCNYTLPLRMETCDLERWSISRAVCTGLCVYMLPILGLIVVLSTLGNAVTLRVMWCNRARRSSINMLITHLAIGDLILTYAHVLVVACWYYTFYWYGGGVLCKIHKYLTVFGDYFLSAMMISIALDRFMAICFPLSRMKSTKRARVMVITSWTLCAIISIPQILIFTVIDETQTVFNVPNFDSGRPQCVDYGFWFDHGGEPMKQVYNLGFVPTAVFLMPLCVFLVAYAAIFINISRSSRFRKSDAQTLTASQVARSQLFNKAKVKTLIMTAVILTTFLINWLPYNVATFLSLFAIDSDPSTTQLLMAFGVSNVVFNPVVYGAFNAASLRQICSKIRSKDRKSSISSRATVVETVNLRSRTLLTVNSNNARMAASVSSCSSNATP

>Xenoturbella_X.boc_g3230.t1_Corazonin_GPCR_homolog_2 (This study – New to Xenacoelomorpha)

MAEPTFSLTPQPLLNGTPDWLESIAVYMDNYEYMLDVYCNGSLETGCTAPVNPVLSAASYAFVAVLCIIVVLSSVGNILTLCVVRRKKDRNSSINSLITHLSIACLLSTYSKVLVDAVYYYTVVWIFGNAMCKVYSYMTMFGDYCMAGMMMCIAIDRFMAICFPLSRVRSTKRTRIAIATSWIASAVMSIPQAIIFEEIDEFVFFIRIPWDSGLYQCKDFIFWRSHGGEHTKKIYTMITQTLLFFTPLLLFLLTYTMIFVQISHSSGSQKSEGRVLTSSQQARYQLFNRAKIRTVLMTATILITFLINWLPYTVGTVVALFYTTPSPMTSSILFCFGISNVFLNPIVYGAFNYRSLSRKKQNDTKTVPTNLYTHSPCARRAAPVNINLRPQSPSVVASDNNSSNDVYRCT

>Xenoturbella_X.boc_g4227.t1_Corazonin_GPCR_homolog_3 (This study – New to Xenacoelomorpha)

MSESDRPNSTIDIELTNDTVNGSNFVIDLTSIKQFLAAYSCSDNFCMGILIVYIVLIVLSLIGNIFIIVSEINNRRKRNTMSTLFLHMAIADLIQTVFIMLVDTIWRFTESWYGGQAFCKIYKFLNMFGLYLATYIVAAVVMDRCVSIWQPLQNARAPRRVKILLTVAWCTSAVLSTPQAVIFDVVYADLRIINRTLTVCGDFHFYSQSSTSPFPLGRKLYSLLVLLVSYVGPTLIMIVLFVLIVYRLTSQRKQKEFCGMNSNDPMMMRRDKLLAKATNRLLTMAVVIVTVFLINWTPYHVGMAMMVSGVELSSVVQHILLMFGSTNCIFNPGIYVIFKYPSFLHGKRSRGSHVDRSCFTSNDEHKNGVRVKKLVSAKSQSTV

**TRH Rc**

>Xenoturbella_X.boc_g6832.t1_TRH_GPCR_homolog_1 (This study – New to Xenoturbella)

MNDSFLDINMTTLNVGTNCCLPVNATSPGHMPRYPEYFTNEETVIHFLIYLVIFILGTIGNIMVILVIVKFRHMQTTTNCYLFSLAVADIIVLVFCMVPSMIENFTIKTAWLLGTVGCVVIVMIQNVGINASSYSIAAFTVERYIAICHPMRAHYLCTPSRAKKIILIGWIFTVLYGASWLFLFTSKTVTFDDGTVIDTCDYKYENNLHIYTSIYLADFIIFYLLPLFMEITMYLMIAKVIYSGDDILNQPKGSRASDQEKRKKLSSSRKQVVKMLVVVVMMFTLFWAPYRICVLYIGLSGHDFTVGFDWFTFFMRAMVYLNSCTNPILYNAMSGKFRMAFKRLCVCEEDDMFQCGRCKKQFTSLVVFMNHKRDECNIRMLQQQQQQQQHQTNTPMQSQLHQSSCESRNNTCTVISESHCTLGLDQGLACSSSISNSTGVVTDTTCLQTFDGLPPSETNPTSTAIHVMETDPSSLTSIKTSSDQMLASSSGVDSTRHILLQDNTMLTLTNTQCIDQSNPVPVSSLFNQVNMLPTSGSTTILQQEPQQQLADGTCIPTSYTYIPLPAGTHQHPASLPVCSVNNTLANIISTTGYKQCPLQSVPQTLTVITSNSDNQPMSVTNSRQTRVSSNVYERRKLKCTYCEKKFVKNFDLQQHLRTHTGERPFQCIVCGRAFAQRSNVKKHMQTHKVWNTSGSLPKEMVIQKTYNTTAAAGCEERPCVDVADASTAVGSTDTVNDNVEEVLSDLIVESSSVGGVKDAATSEDGGASAKLIVNNGFMCRYCNAHFTYYYDYKKHMTKHKDEQAESVDTA

>Xenoturbella_X.boc_g6834.t1_TRH_GPCR_homolog_2 (This study – New to Xenoturbella)

MGTVAEAISGLLAVGSHATPDDNASIVDQSTSIPLPDMSPQHTSIIQNTSDMPTSLLPEYYYIPYQVVATALFGLIWIEGVIGNSLVIAVVIFTKRMQTPTNCYLVSLAFSDLLVMVVGVTPNFIEHFTPIDSWSFGRVGCLLVVVLQNAGINGSALSITAFTVERYIAICHPMRAHTMCTAGRAKKIILTLWAINTVYCSAWFFLFDTARIAYADGTSIEICDYKEMDPAAYILIYMADFTLYYMAPMIMATVLYAMIARALYATTNVPRRSTRRRCDRTSVGNGGDKALKLKKSNAGISSRKQVIKMLIVVVLLFGVLWAPYRFYVIYIFLDPNPPATDNIWVVLFLRTMVYINCAINPILYNLMSMKFRKSFARLCRCRVSNKRSSTYRLTTMNSAVTEESNAT

**TRH ELFGa Rc**

>Xenoturbella_X.boc_g9713.t1_TRH_EFGLamide_GPCR_homolog (This study – New to Xenoturbella)

MSDIDTGNSSVGSTAYYNGSYENQCIPNITADQMYAPLSTAITSTIMHLVIGIIGLTGNALVIFVVVRSRRMQTPTNCYLVSLAVADFMLLTVTNLANLPQNFLIQGHWPYGLAGCKSLMVLAYLGADASCLSVTAFTIERYIAICHTMKAQTICTVKRAKITIASIWVFCLLYTSIYGYFSVYYPLCLNDETTYIWVCEPVDPQSLKIVVIADLIIFYLTPLFTSFILYALIMMALFSSDVPRNNKAIEGKWKVVRMLFLIVLIFALCCTPTRVGEAIGYYGTHIPVEYSQMFNVLYLVNSAVNPILYNIMSEQFRRDFKRVCCGKRPDKLNWHRTMTSFVSKSTVTDSNV

**ETH Rc**

>Xenoturbella_X.boc_g6882.t1_ETH_GPCR_homolog (This study – New to Xenoturbella)

MTDGLLTDEDVFLGRYVPAVNSTFANDSYQSWFNVCQNAPEIEGAIQDLCCGTGSAPEFPYHLPVTTLAVATFIYIIIFIFGILGNSLLCFVIWKTSDMRSSTYVLLGNLGVADLLVLVFSMPSKLLEIYRNLPWMLGRAMCVIVGSMETISTQTSVLTMLVIASERYCAICHPLKVQYVCTVSRTVKVIVCVWLFAILTTIPYMVHSSIQIYWEPINGYCYDECKNTLPDMSNLSLQILFFFIPLILLIAMYSAIARQLLNDKYTQEAVSSENRAKNMKTRRQVIGMLAAVTTIFFVCWLPRRIINLLLVYKRDALIWLDYNGYHHLEMVCNILVYINSAINPILYNMISSKFRAAFMTAMGCRQKRRFRRSGSTVTSYTSVTTYKTNSNQSMNPGARTPNWRHDSGNHNLNVASSYA

**Orexin / Allatotropin Rc**

>Xenoturbella_X.boc_g9404.t1_Orexin_Allatotropin_GPCRlike_homolog (This study – same as 21676.1 – Thiel et al. 2018)

MNALGILPGVFSDSESADTDVTLDGAVPMTTKPSRNTTVVNGVNMTSFPAMSLDYAESQDYSNYSMDSWRDDDFLWDYYVRPNKCEWILISAYILVFCLAVVGNALVVFVVFKNRHMRTVTNFYIVNLSIGDLIVMIIVLPPTLVVDVTHTWFLGDILCKVIPYLQQVAVSVTVLTLSVISLDRFYAICHPLKFKSTTSRAKHYIIAIWAVSLVVTAPFPVVMRTNVFLTNPDSVMFTECKEFGWPPGPGRVVYHLFLVSVTYFLPLSLMGAVYYKIGRRLWTIELPGLISDGSSYRNGISANKASDAQIQSRRKVAKMLLVVVIIFALCYLPLYVLQFIRAVNLEQSLMKRPGFNIWALVAHWMCYLNSAINPVIYNFLSGKFRSEFRAAFQSCRECPRYANRRKQSLSWTRTTSFKSRSEMTMTSFRDGRRVNSVTTA

>1572_Xenacoelomorpha_Xenoturbella_bocki_21676.1_Orexin/Allatotropin_related_rhodopsin_type_neuropeptide_GPCR_5prime_partial (Thiel et al. 2018)

EWILISAYILVFCLAVVGNALVVFVVFKNRHMRTVTNFYIVNLSIGDLIVMIIVLPPTLVVDVTHTWFLGDILCKVIPYLQQVAVSVTVLTLSVISLDRFYAICHPLKFKSTTSRAKHYIIAIWAVSLVVTAPFPVVMRTNVFLTNPDSVMFTECKEFGWPPGPGRVVYHLFLVSVTYFLPLSLMGAVYYKIGRRLWTIELPGLISDGSSYRNGISANKASDAQIQSRRKVAKMLLVVVIIFALCYLPLYVLQFIRAVNLEQSLMKRPGFNIWALVAHWMCYLNSAINPVIYNFLSGKFRSEFRAAFQSCRECPRYANRRKQSLSWTRTTSFKSRSEMTMTSFRDGRRVNSVTTA*

**Cholecystokinin Rc**

>Xenoturbella_X.boc_g5458.t1_Cholecystokinin_GPCR_homolog_1 (This study – same as 21634.1 – Thiel et al. 2018)

MNANNGTVYQLKGSGEAEPTPEIAVCVLVVSVNVSVATLIVIALERYFAICHPLKSRVWQTKNHAVVAISVTWLCSLVVSSPILVVAKSRNYQPPSMEPKYNCREDWEGIGSPTTYTICMFTIQLVAPVLVMSWAYAMIIRTLYVAGQLDKPSHDHSSGSKKSDSVDYNHLAVTSTSAASKRSENRMRSTGNSESAVNAKRNLVKMLVVIVLLFFVLWLPMHVMNMWLIFDQMTARCYITALVAAIFHILTYISCCVNPIIYCFMNKRFRQSFVSLLCCVPKKYITSQSTVSKGNQYQKWSANRKTVESQVASDSV

>1571_Xenacoelomorpha_Xenoturbella_bocki_21634.1_CCK/Sulfakinin_related_rhodopsin_type_neuropeptide_GPCR_complete (Thiel et al. 2018)

MAYPNRTMTPSQPVGFHGNSTNSTFSPDFTMDAMMMYFANCPKHQIHDAVKITLQVIVFCLATVGNFLVVLTLVMNKRMRNVTNLFLLSLSISDLMMVVVCMPINLIGQTLKNFLFGEFMCKLVTFLQIVSVNVSVATLIVIALERYFAICHPLKSRVWQTKNHAVVAISVTWLCSLVVSSPILVVAKSRNYQPPSMEPNYNCREDWEGIGSPTTYTICMFTIQLVAPVLVMSWAYAMIIRTLYVAGQLDKPSHDHSSGSKKSDSVDYNHLAVTSTSAASKRSENRMRSTGNSESAVNAKRNLVKMLVVIVLLFFVLWLPMHVMNMWLIFDQMTARCYITALVAAIFHILTYISCCVNPIIYCFMNKRFRQSFVSLLCCVPKKYITSQSTVSKGNQYQKWSANRKTVESQVASDSV*

>Xenoturbella_X.boc_g5167.t1_Cholecystokinin_GPCR_homolog_2 (This study – same as 20472.1 – Thiel et al. 2018)

MGKAVFLLLAVTFHGIVALDESLNSTLLNETLFDPFRPWPEKRGVSLTDMGIGEIVGVSSIFILSVTGNTLVIITIVFNRQMRNVTNIFLLSLSVSDLLYALMCMPPTLIGHILKKFIFGEAMCKLVPVLSNANVNVSAFTLLMIAVERYFAICQPLRSRAWQTRGHAYVAITLTWIAGIVIASPLLQYQVHRRYPDGSFRCGEIWSSKLSMMLYTFVLFAVQLLLPFLVMAVAYTLIVRQLFQGVVVREDPKKEPRAGLVTNTGDGPSDGQTVGEARKWRKKGKKADKPTSMMRSTDSDRALKTKRRLVRMQIVVVILFVVCWAPVHAMAIFSTLHPIAANRIMFKYSVWLQLLSYCSACVNPVIYCFMSKRFRKNFCDILRCRNVRCMRNRPRNTRQYTVSTKTSQMWSKSSTSVL

>1569_Xenacoelomorpha_Xenoturbella_bocki_20472.1_CCK/Sulfakinin_related_rhodopsin_type_neuropeptide_GPCR_complete (Thiel et al. 2018)

MGKAVFLLLAVTFHGIVALDESLNSTLLNETLFDPFRPWPEKRGVSLTDMGIGEIVGVSSIFILSVTGNTLVIITIVFNRQMRNVTNIFLLSLSVSDLLYALMCMPPTLIGHILKKFIFGEAMCKLVPVLSNANVNVSAFTLLMIAVERYFAICQPLRSRAWQTRGHAYVAITLTWIAGIVIASPLLQYQVHRRYPDGSFRCGEIWSSKLSMMLYTFVLFAVQLLLPFLVMAVAYTLIVRQLFQGVVVREDPKKEPRAGLVTNTGDGPSDGQTVGEARKWRKKGKKADKPTSMMRSTDSDRALKTKRRLVRMQIVVVILFVVCWAPVHAMAIFSTLHPIAANRIMFKYSVWLQLLSYCSACVNPVIYCFMSKRFRKNFCDILRCRNVRCMRNRPRNTRQYTVSTKTSQMWSKSSTSVL*

>Xenoturbella_X.boc_g5280.t1_Cholecystokinin_GPCR_homolog_3 (This study – new Xenoturbella sequence)

MVEPVTILADTFVSTVPSTVSGADESNGDKIDVQVSLNAALSILILVVSIFGNTLVIMTMVVNKQMRVVTNLFLLSLAISDLMFTVVCIPITAIGQILEEFIFGEVMCRIAPFLQGTTVNVSVLTMVVISLERYFSICQPLHARSWQTKRHAFVAILTSWLVGLVAATPIIFLQTHSVYKDGKYSCRESMGDFQRSYTIVLMLGQLVIPLIVMTVTYTLIILELHAVSSQSTRRKQAGRPPTKETLSYPDDNGDLAKPLKRKHKKKIRFKNNGKPTKQNHANQHQLQQQKLSWNESNLKNKAKLIKMLIVVVVLFFLCWTPVHVINIWFSFDPPGAMKVLSPYSLLFQSMAYSSACVNPITYCFMSPRFRRSFFSLVICCSRQSYWYKRQRRNSPRPKSTQISMQSRPTNATLTLTPIISTGVDQKSRGTGH

>Xenoturbella_X.boc_g13452.t1_Potential_Cholecystokinin_GPCR_homolog_4 (This study – Potentially new Xenoturbella sequence)

MEPTPAMSSPVTSSSSVQQVSPYSWNITNPVISSYILMFDGHYFDYTQINVVRLLFIISYSVIIFLSFFGNIMVIWTVWRNKHMHTVTNCYIVNLAISDVLMSSLIMPQSLIQYAVESESFLSVQLCMAIYYLEGVFVSVGVLTLEAVSLERYFAIVHPLNSIKLHSAKRAKKILVVIWVIPLLAFIPVLNQGKIGVFNMVSDWGSIYRMICHVDFQNISLYFHLNNFEFGYTFFLFMMLYLIPLLFTGITCVMIVRHLLFKDRTDSGDLLRGQAATLRAEENRKKVAKMVIAVVIAFFIAWTPFYIYHLRDATSPNNRMHVKSHYFFVLLLIKFLAYSNSSVNPMIYMIMSKRFRWGFIDLFKLVFCCVRKRSSANSSTQSDQSRHSYEMRDTIADRKKRARFLKSPVETPSQDSSSGFVDSDPNLRPVKLRYADVVSHYENGQRKVGNVICHPNGNVEYVPSASRNTNPLGPSKQKLDGQSCCFGNSHKSNGSYHYTKNNPDFDNGRNAKRQRGHAKPEQKAVGQKEIAEIAQKLSETSSENNTAVKYSARHHLHQPCEMKTYATSQSEGSPLTYDPQNTELLSFGKSCASTPAIPDCQRFHNIPAIRIVDNFNKEPDLEGYYDFLWSAPENSSYRSYINRSIPSLQNNVVPSAHNVRSHVVSRPDGTITDQLQTCLRQCTDNCVCSGFSRNKFKSTNVDMIKSTETSPTAGRRGHSFSF

**Gastrin/CCH/Neuromedin Rc**

>Xenoturbella_X.boc_g7970.t1_CCHa_Bombesin_Neuromedin_B_GRP (This study – New to Xenoturbella)

METNESWFAYNDTYLAQFNLTLFDGPRLSSKDLEDIILGPDNQVSIVVYVLIGIVGIVGNSAVVRIVLTNRLMRNVPNILITSLALGDILLLVVNIPIKIRENVTVSWTLGAFVCKFSVMMQIVSMSVTILTLVALSADRFQAIMCPMNIHASNLMTRTYAWAIGIWVISFLLAIPDAINTETGPYLYGIGNDTLPVKGHMVYVNYQVVPLTYCSPFPRSSQRDNLYPMLYFVVRFVLLFIFPLAIIGTFYSVIAQHLFMSSTTLPGEARATKSDHKQLKARKRVAKTVLVFVAMFVVCVLPDHIYQLMMWSDNLPISGSYYAVFTMAFRYISIYLLYISSCSNPIVLYFLSNNFKQYFNRYLFCCRRPRKKPIRNFDSESTMRGVTTSLSTRTIVDAV

**PTTH Rc**

>Xenacoelomorpha-Xenoturbella_bocki-g11642.t2-PTTH-Rc (This study – New to Xenacoelomorpha)

MIVMPTVLVIFICVGVGFWYIRIRRRPVFSCSSINENYYIPSPERDRETPDHWEIPHVGVELGELLGQGAFGQVYKGKVRGSLLYHKTSFTVPIDHGDDTFIDVAVKILKEFASDTQKQEFRREIKLMKDMGHHNNVVTMLGCCTLRDPICLIEEHLPNGDLLNYLRTQRKGNILNEDMLVKPCFGEETAHISEPKYINIHNQEPKPPVTQDDMLSMIRQICLGMEFLSQKGFVHRDLAARNCLVGYNSVVKIGDFGLTRYIYDDKVYCNRRGGKLPIKWMSIEAIMDQVFTTQSDVWSFGIVIYEVMTLGSSPYPAISNVELLSLLLCGYRMPSPPGCPDEIYNLMLDTWKENPDGRPNFTDLKRSIEGLMCGRLKYMDFADTGSELVVYTNIGSMDNENSSSEANSETKTMVTNL

**ILP Rc**

>Xenacoelomorpha-Xenoturbella_bocki-g2891.t1-ILP-Rc (This study – New to Xenacoelomorpha)

MKPVEHYANASLTGNVFCGTVSVRNNASQLKQLENCTVIEGSLQLMLMDYTEWDDFKDLSFPHLVEITDYFVLFRVTGLKTLKYMFPNLAIIRGHDLFASYAFIVYEMVSLEEIGLYSLQHISRGGVRLTENRNLCYLETIAWNQITSDGSDASSLDNKRVEECADTCRPDWSEGESVCRYWHQNIRNLCWSQDKCQIVCPDECPGNCRWPDRVSVQECCSDFHQRRCVRAADCPAEYKLFEGVCRYGCPGNYMPEVIEIEFGIVRETQACIPCTGTCDKTCFSTTVNTIENARKLKGCTIIDGSLTISIQSGHNIVNELDENLQLIRVVEGYVSITQSHSLVSFSFLKGLETISGKDLKYDVYALYVMNNDNLETVSLGGSNNTVIENGKVSFHYNPKLCLNEIYYLTSNLNMVEGVDFDDRDIGVNSNGDKAGCSHTTLNVTAQTYGTSMLIKWPPWYPDDSRALLNFVIYYREAPEQNVKMYEGVDACGTNPWITDEIPPKDNVAALTGLKPWTQYAFYVKSYLVAGLSYKSALSDITYATTQPAVPGPPQNLRGAAMSTSELSIQWDLPADPNGDISHYIVKWRKNTVNTTILDLRNYCSDDKFSFPEPSESIYYTIDRNDEDDSNLTETWLVDGEKRCSCPKTEEELRAENLEREMEIMFENFLHNSVYIKLDPDSMIMKRSIPDSYYTTMMSSGNQTVTATPLPTVLPPLSNDTAPTTAPENMIRVNLDREIRLTNLSHYTQYVIYVFACNQVNCSTMPQQTAITTHPDQRADNVVGKVTGKVINNRTHEVLLQWREPKFPNGAIVYYQLMYNSIENGILQDYIETICIRHSDYVKYNGGFKLKELEPGNYSFQIRATSLAQFGDYTDKMYLYVPDIKEDNSNKFKVVLILVTVSALFLIFCVGLTVYIYLRKRMVNRAAQNGVLFASSNPEYVSCSYVYVPDEWEVLRDHVTILHELGQGSFGMVYEGIAKDISDDYPGTMSVAIKTVNETASVRDRIEFLNEASVMKAFTCHHVVRLLGVVSIGQPTLVVMELMAHGDLKTFLRSHRPDEDGTRSDMQPDLKQFLQMAGEISDGMAYLSARKYVHRDLAARNCMVAVDFTVKIGDFGMTRDIYETDYYRKGGKGLLPVRWMAPESLKDGVFASQSDVWSFGVVLWEMATLANQPYQGMSNEQVLKFVMKGGTLDKPDDCPDKLFELMRLCWSYNLRARPTFIELVEILEPDLKESFQNVSFYHHRIITQNETVPPQTESHYDTNDSPYDNPYDNRRITPNSTHPANGGMSLKSSDTTAC

**Relaxin Rc**

>g5386.t1_Xenoturbella_bocki_Xenacoelomorpha_Relaxin-Rc (This study – New to Xenacoelomorpha)

MVGNLCVMIGRILMKPENRVHSLVIKNLCAADFLMGVYLVIIGTHDVMYRGVYNRNSLKWLSSWPCHLAGITAMISCEVSVLILTFMSIDRFICIAYPYFNKRHEMKQTFVTVGMIWVSGVVFAVIPFSSQEYFGNFYGSNGLCFPLHIHEAYLEGWQYSFFIFLGLNSASFFTVAVCYVAMFFSIQRTRQTCPQINLKGEYSFAQRFFVIVLTDFLCWIPIAIIKIAALFYVHIPTDVYAWVAIFILPINSCINPIVYTMSTNVFKQYFGRSVTMIRSKFGRSSGQNGGPHRRILKKMSTFVSDVNSIGNTCETDVHVPSSRAVDNCPQSKLSLPLLETDRLHVESGGAEQTMATSQDL

**Bursicon Rc**

>g5048.t1_Xenoturbella_bocki_Xenacoelomorpha_Bursicon-Rc (This study – New to Xenacoelomorpha)

MVAVFGNACVIIVNILSKVKMDVPRFLICNLACADFIMGIYLGFLAIVDASTIGHFQQHAVQWQTGPACPIAGFLGVLSSELSVFILAIITLERFYAITYAIHLNKRLTLRQAIHVIAGGWCLSILIATLPLFKISDYQEFAVCLPFPLPFPIPAEKIASRVYIVTLMVSNSAAFIVIVFCYTCMYWSIRGSHAWNSKDSRVAKRMALLVFTDFCCWAPITVCSIAETFGYDLISLEGTKILTIFILPLNSCANPFLYAISTKQFKKECVGVCRRIEEKHIDGSLWVSQMKPNNIFRELARCACFRRGGTTDRDPALVYNCPPAVGLQLTNLSASSKRPSNAGDASMGGGGGLFESIPTPRLNPASLDNSSSANTSDSLNKPVGVKIEITVIDHTYPCAPRGHLTPGPGAGDSQTGAAGPSSQRLSVPHHPDEASITTIETDFTELDGDGAGASPEGFGDGPTPVPSGGEGGQSPVPRGRTNLISLKMSERADDGGSSRSLEIEADYSSSSSEYSAREPAPSRPRANTDSTLVAMLTRIGFSKRRSLRVKVKIGSGSGPEMECPWVKICTGQSRLNSTVGPWVKICTGQSRLNSTVGPWVKICTGQSRLNSTVGPWVKICTGQSRLNSTINVAVGKTASQSSTYSTLDAGNAVDGVITDSSDAALAHTDPLQSSTVHWWVVDLGYKEDIATVAVYQRGAPHSHRMLGSEIWVGGEANPDKYTDNVLCGTMDTNNVVDGGVTTIECDKPGRYVMMVKDFGLKDGDTCFNFFEVEMNKSGGAAEKAKRRVTYMLITVTILYLLLVGPYYMHRMVMYHTGALQVWPWRYAWLNDYDVAYWVPYEGFYNIESILQGLLLITMVMTGQITMVMTDQITMVMTDQITMVMTDQITMVMTDQITMVMTDQITMVMIDPHGND

**Glycoprotein Hormone Rc**

>g13796.t1_Xenoturbella_bocki_Xenacoelomorpha_GPA2-GPB5-Rc (This study – New to Xenacoelomorpha)

MIFVHYRNLSNNTNLQHIADDAFAGLEKELRVLSLNATAIRTLPTEGLDSITEFYMAHTETMVSLPEPLFHLKNLKFTVLTYFSHCCAFDRPPSGAGDIDYISYQAATNMTPRLLPTNCSNYDDPQSTATVGANGHTIVPITDANSDDGVFGDIIPETNRVDNTGDEYRPNTPPSRDNLPTVDPNASPAAATPSSDQMFLPPRGKPQCFPRPDPFNPCEDVIGDQVYLRVIVWVVVLTAMTGNAAVLVVLLSNYSKMNVSRFLMCNLAFADFCLGLYLMIIASVDAYTRENYYNYAVHWQYGTGCRVAGFLTVFASELSIFTLTVITLERYIAIIYAMHLTRRLRLKMAAKVMFCGWMFSIFLATLPLFGVSSYEKASICLPLDASELNGKVFINSMMFVNLIAFLIICSCYIQMYLAIRNPAMVTGKNDKTVAKRMATLVFTDFACWAPILFFAFTAMVLPESLISVSKSKILLVIFYPLNSCANPFLYAIFTKSYKRDFFTMASRFGLFKKKAMKYNGTITSNPNRSRTYSSGIRYGRPRYSANRHGSDGSVLTTSLAGSAENSGSCSPLPSPMTYYRDSPIPIHRLTVHISLPTLTMNRSNSDCSRIVSEMDPLGVGKMMPRTNTLDNSSISAAGIH
