# Supplementary figures and images for: Insights into early animal evolution from the genome of the xenacoelomorph worm Xenoturbella bocki
Source: eLife. 2024 Aug 7;13:e94948. doi: 10.7554/eLife.94948 (PMC11521371; doi:10.7554/eLife.94948)

# ILP-3 Bombixin

## IGF

## dILP 6/8

## dILP 7

## Multinsulin

# Insulin 4/5/6 GSS

## Octinsulin

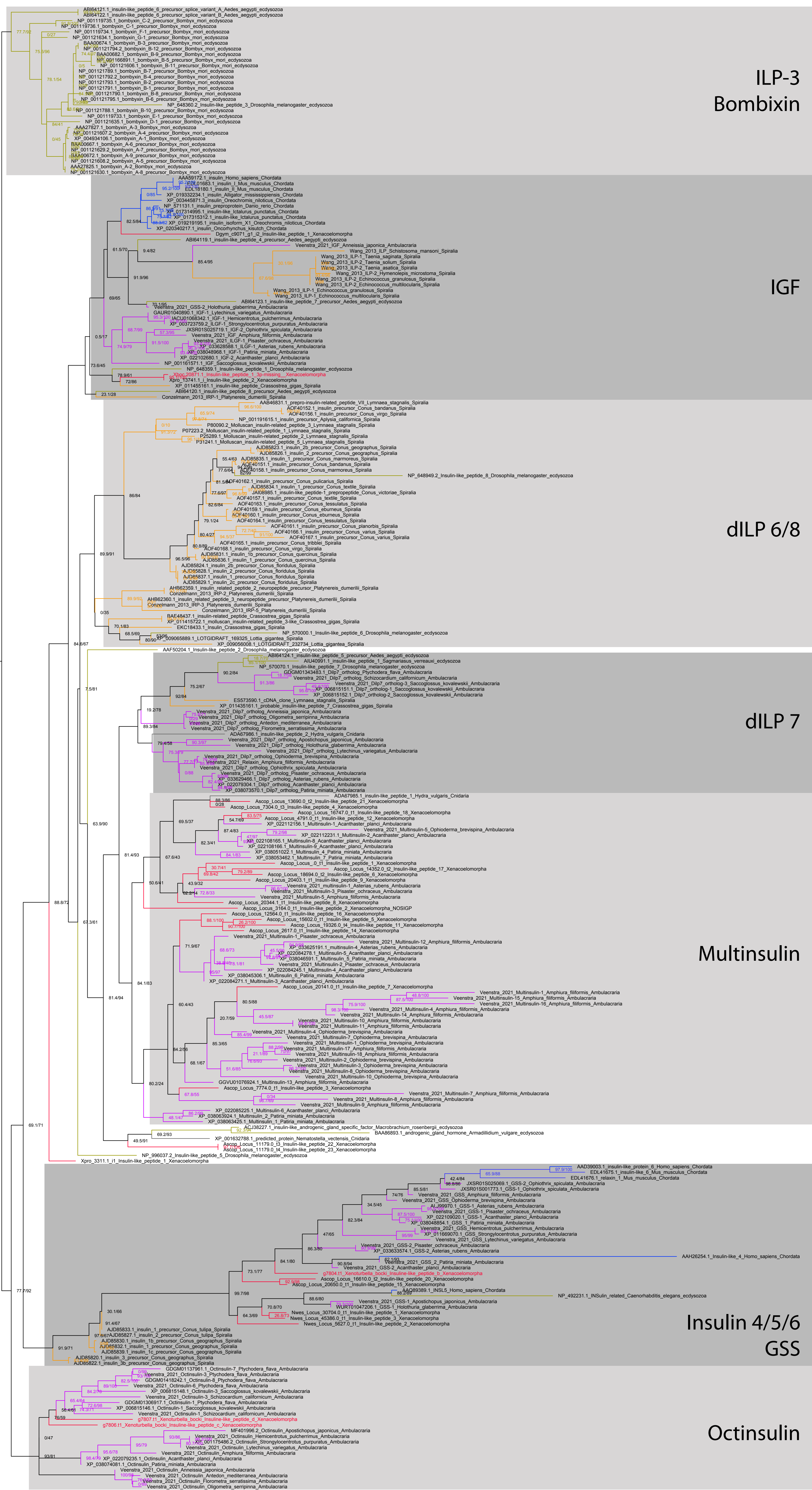

Supplement: Supplementary file 2. — Tree is calculated from concatenated alignment of A and B chains. Numbers represent support for nodes calculated using 1000 ultrafast bootstrap replications and 1000 SH-aLRT replicates, respectively. Scale bar unit for branch length is the number of substitutions per site. Branches are colored according to the phylogenetic position of the organism from which the sequence originates: red, Xenoturbella; pink, Ambulacraria; blue, Chordata; orange, Ecdysozoa; green, Ecdysozoa; gray, Cnidaria. dILP, Drosophila insulin-like peptide; GSS, gonad-stimulating substance; ILP, insulin-like peptide; IGF, insulin-like growth factor. Radial version of this tree is presented in Figure 8—figure supplement 2. Sequences are available as Figure 8—source data 1; alignment and IQTREE tree files are available at https://doi.org/10.5281/zenodo.6962271. [file elife-94948-supp2.pdf]

GRL-101

dILP7/Relaxin R

GPA2-GPB5 R

Bursicon R

GPCR

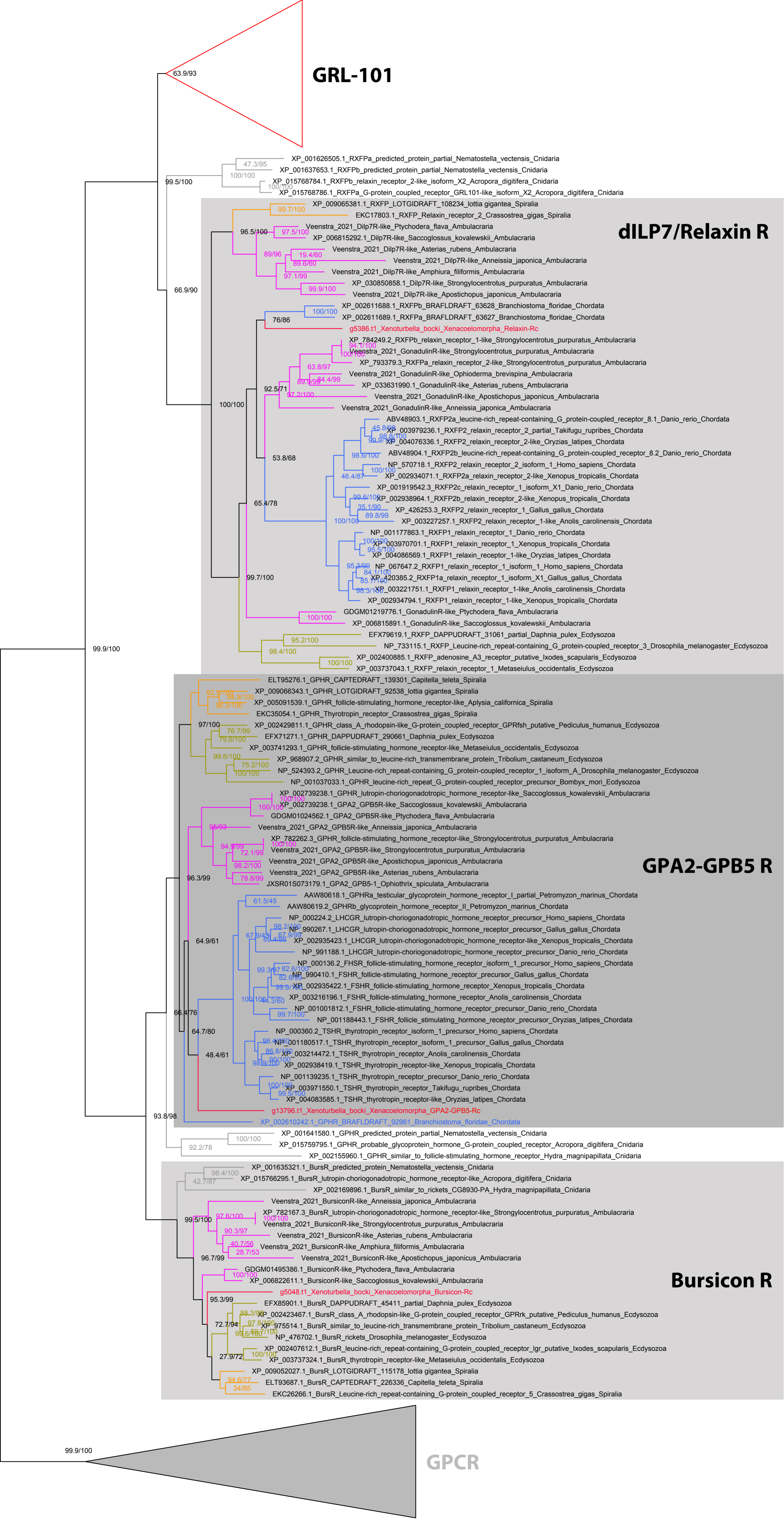

Supplement: Supplementary file 3. — Numbers represent support for nodes calculated using 1000 Ultrafast bootstrap replications and 1000 SH-aLRT replicates respectively. Scale bar unit for branch length is the number of substitutions per site. Branches are colored according to the phylogenetic position of the organism from which the sequence originates: red, Xenoturbella; pink, Ambulacraria; blue, Chordata; orange, Ecdysozoa; green, Ecdysozoa; gray, Cnidaria. Collapsed group colored in red indicate that they contain at least one X. bocki sequence. GPA2, Glycoprotein Hormone alpha5; GPB5, Glycoprotein Hormone beta2; GPCR, G Protein-Coupled Receptor; GRL-101, G-protein coupled receptor GRL101. Circular version of this tree is presented in Figure 8—figure supplement 3. Sequences are available as Figure 8—source data 2; alignment and IQTREE tree files are available at https://doi.org/10.5281/zenodo.6962271. [file elife-94948-supp3.pdf]

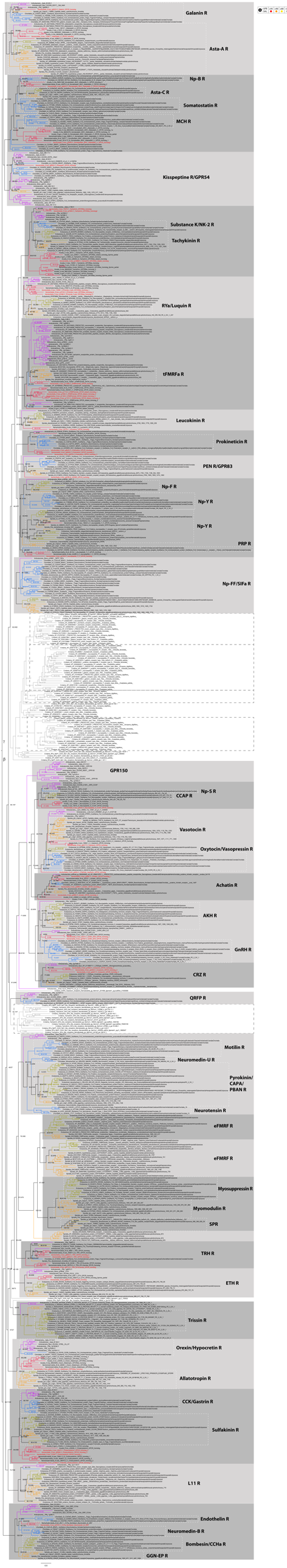

Supplement: Supplementary file 4. — Numbers represent support for nodes calculated using 1000 ultrafast bootstrap replications and 1000 SH-aLRT replicates respectively. Scale bar unit for branch length is the number of substitutions per site. Branches are colored according to the phylogenetic position of the organism from which the sequence originates: red, Xenoturbella; pink, Ambulacraria; blue, Chordata; orange, Ecdysozoa; green, Ecdysozoa; gray, Cnidaria. White boxes with associated name highlight groups of annotated sequences. AKH, adipokinetic hormone; Asta-A, Allatostatin-A; Asta-C, Allatostatin-C; CAPA, Cardio acceleratory peptide; CCAP, crustacean cardioactive peptide; CCHa, CCHamide peptide; CCK, cholecystokinin; CRZ, Corazonin; eFMRF, ecdysozoan-FMRFamide peptide; GGN-EP, GGN excitatory peptide; ETH, ecdysis triggering hormone; GnRH, Gonadotropin Releasing Hormone; GPR150, G Protein-Coupled Receptor 150; GPR54, G Protein-Coupled Receptor 54; GPR83, G Protein-Coupled Receptor 83; MCH, melanin concentrating hormone; NK-2, Neurokinin 2; Np-B/W, Neuropeptide B/W; Np-FF, Neuropeptide FF; Np-F, Neuropeptide F; Np-S, Neuropeptide S; Np-Y, Neuropeptide Y; PBAN, pheromone biosynthesis activation neuropeptide; PEN, neuroendocrine peptide PEN; PRP, Prolactin releasing peptide; QRFP, Neuropeptide QRFP; RYa, RYamide peptide; SIFa, SIFamide peptide; SPR, Sex peptide receptor; tFMRFa, trochozoan-FMRFamide peptide; TRH, thyrotrophin-releasing hormone. Circular version of this tree is presented in Figure 8—figure supplement 4. Sequences are available as Figure 8—source data 2; alignment and IQTREE tree files are available at https://doi.org/10.5281/zenodo.6962271. [file elife-94948-supp4.pdf]

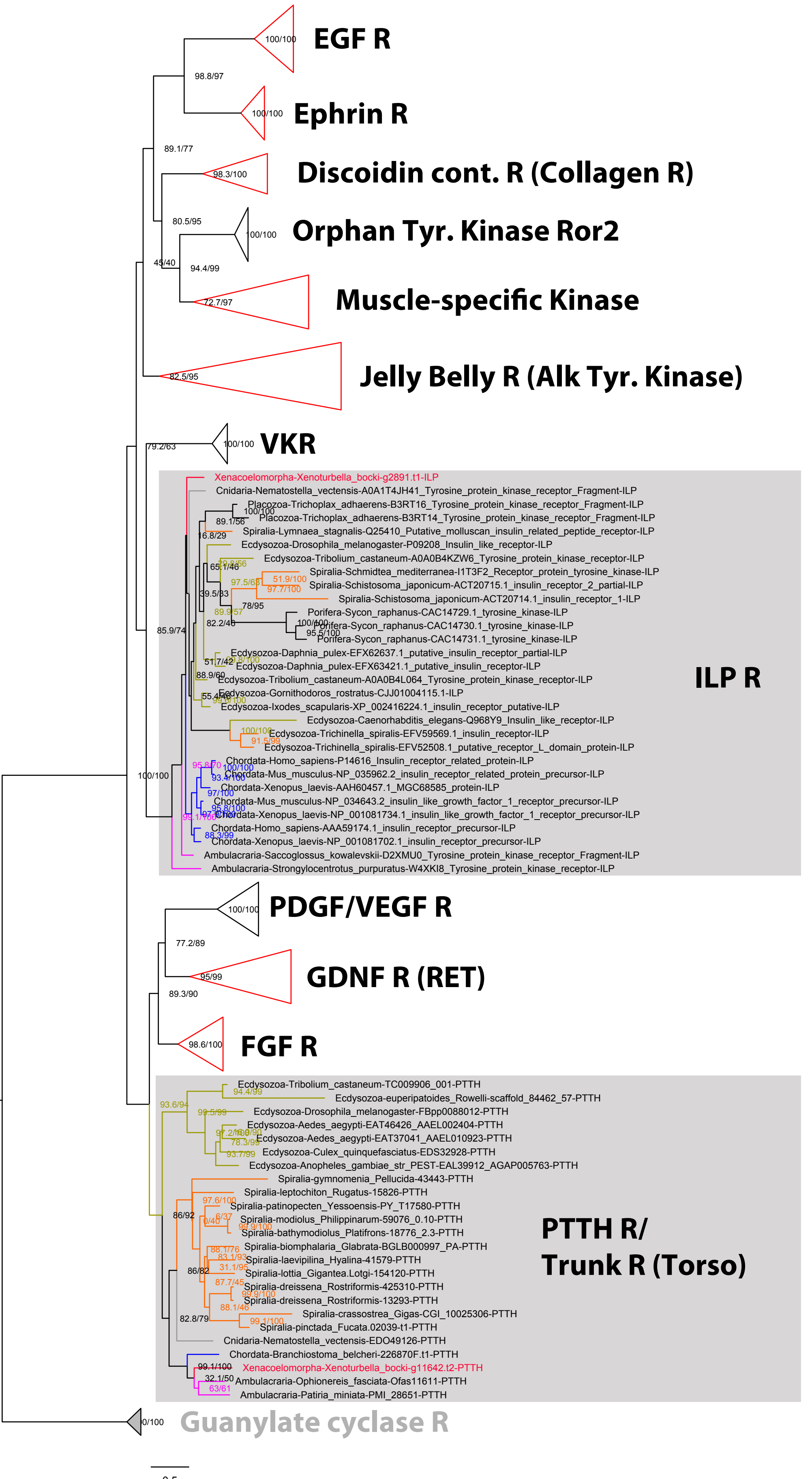

Supplement: Supplementary file 5. — Numbers represent support for nodes calculated using 1000 ultrafast bootstrap replications and 1000 SH-aLRT replicates respectively. Scale bar unit for branch length is the number of substitutions per site. Branches are colored according to the phylogenetic position of the organism from which the sequence originates: red, Xenoturbella; pink, Ambulacraria; blue, Chordata; orange, Ecdysozoa; green, Ecdysozoa; gray, Cnidaria. Collapsed group colored in red indicate that they contain at least one X. bocki sequence. EGF, Epidermal Growth Factor;Discoidin cont. R, discoidin domain-containing receptor; Orphan Tyr. Kinase Ror2, receptor tyrosine kinase-like orphan receptor 2; VKR, Venus kinase Receptor; ILP, Insulin-like peptide; PDGF, Platelet-derived growth factor; VEGF, Vascular endothelial growth factor; GDNF, Glial cell line-derived neurotrophic factor; FGF, fibroblast growth factor; PTTH, Prothoracicotropic hormone. Circular version of this tree is presented in Figure 8—figure supplement 5. Sequences are available as Figure 8—source data 2; alignment and IQTREE tree files are available at https://doi.org/10.5281/zenodo.6962271. [file elife-94948-supp5.pdf]
