# Supplementary material for: Insights into early animal evolution from the genome of the xenacoelomorph worm Xenoturbella bocki
Source: eLife. 2024 Aug 7;13:e94948. doi: 10.7554/eLife.94948 (PMC11521371; doi:10.7554/eLife.94948)
Supplement: Supplementary file 6. — Numbers represent support for nodes calculated using 1000 ultrafast bootstrap replications and 1000 SH-aLRT replicates respectively. Scale bar unit for branch length is the number of substitutions per site. Branches are colored according to the phylogenetic position of the organism from which the sequence originates: red, Xenoturbella; pink, Ambulacraria; blue, Chordata; orange, Ecdysozoa; green, Ecdysozoa; gray, Cnidaria. White boxes with associated name highlight groups of annotated sequences. DH31,diuretic hormone 31; Np-RB1, Neuropeptide receptor B3; Np-RB4, Neuropeptide receptor B1; PDF, Pigment-dispersing factor; CRF, Corticotropin-releasingfactor; DH-44,diuretic hormone 44; PTH2/3-R,Parathyroid hormonereceptor2/3; GIP, Gastric inhibitory polypeptide; PACAP, Pituitary adenylate cyclase-activating polypeptide;VIP-R,Vasoactive intestinal polypeptide receptor; GHRH, Growth hormone-releasing hormone; PTH, Parathyroid hormone receptor; SCTR, Secretin Receptor. Circular version of this tree is presented in Figure 8—figure supplement 6. Sequences are available as Figure 8—source data 2; alignment and IQTREE tree files are available at https://doi.org/10.5281/zenodo.6962271. [file elife-94948-supp6.pdf]

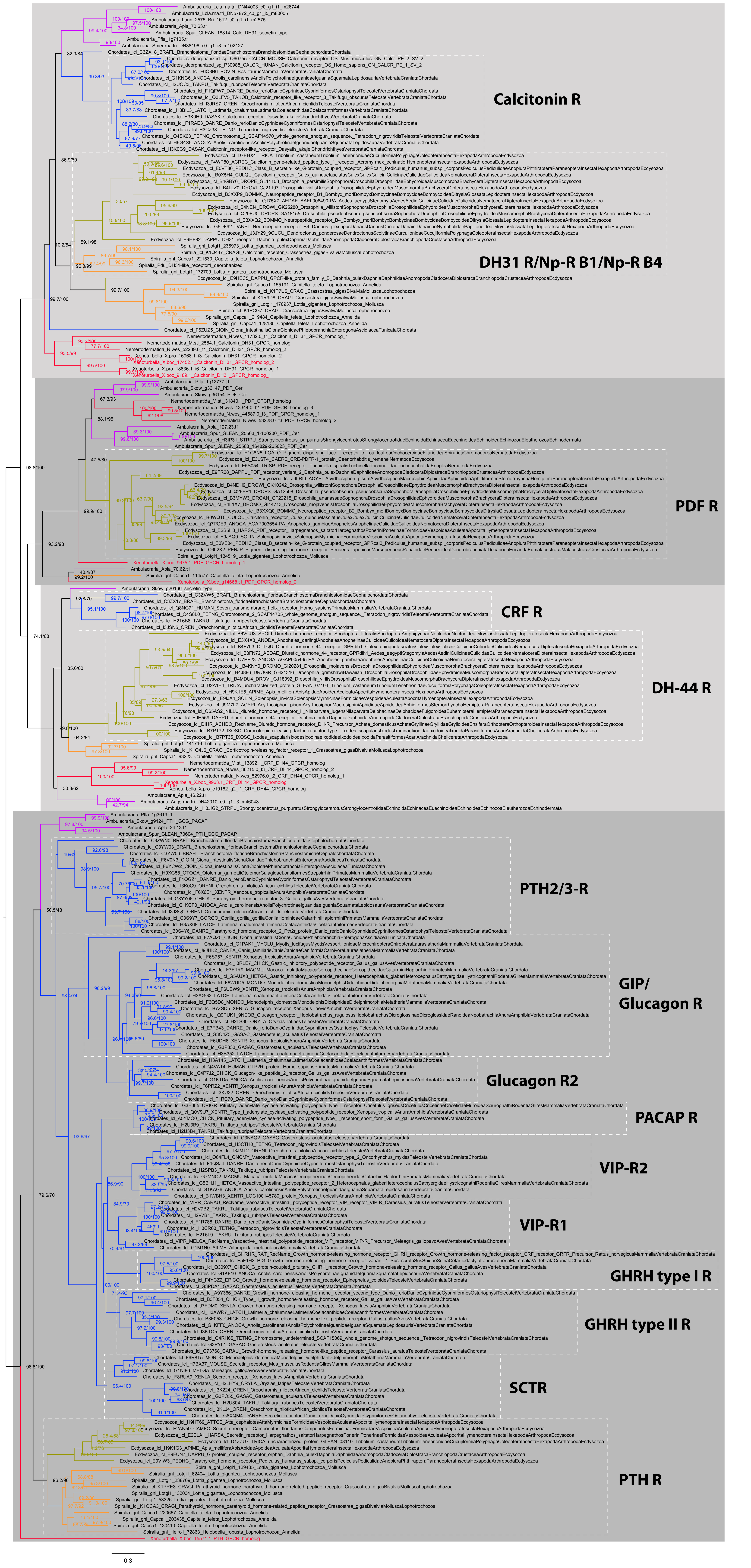
Calcitonin R
DH31 R/Np-R B1/Np-R B4
PDF R
CRF R
DH-44 R
PTH2/3-R
GIP/
Glucagon R
Glucagon R2
PACAP R
VIP-R2
VIP-R1
GHRH type I R
GHRH type II R
SCTR
PTH R
0.3
