# Supplementary material for: Insights into early animal evolution from the genome of the xenacoelomorph worm Xenoturbella bocki
Source: eLife. 2024 Aug 7;13:e94948. doi: 10.7554/eLife.94948 (PMC11521371; doi:10.7554/eLife.94948)
Supplement: Supplementary file 7. — The alignment highlights the presence in all sequence of aconserved ‘PPNPCP’ motif. X. bocki sequence is highlighted by a red dashed line. Sequences are available as Figure 8—source data 1; alignment is available at https://doi.org/10.5281/zenodo.6962271. [file elife-94948-supp7.pdf]

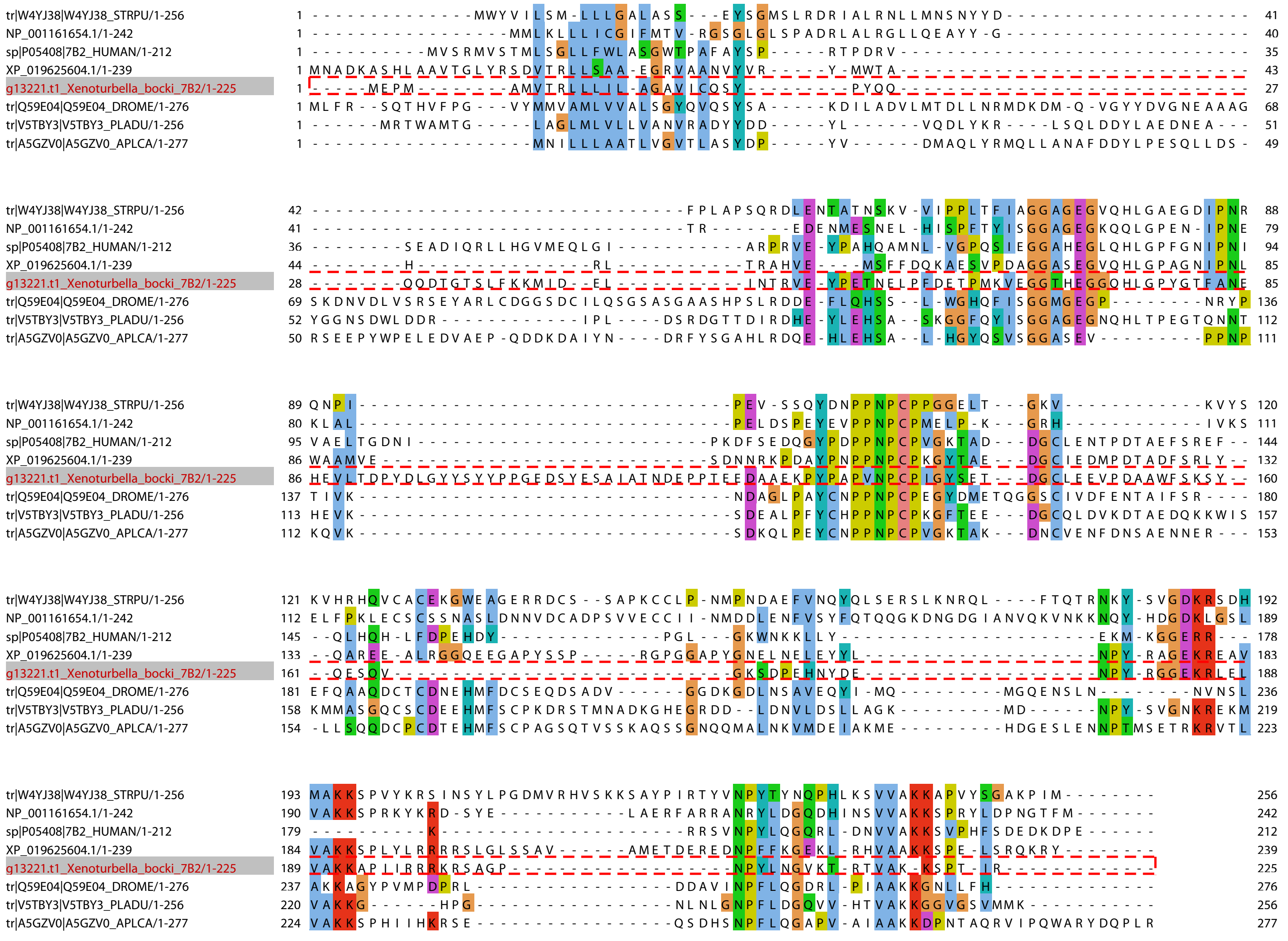
tr|W4YJ38|W4YJ38_STRPU/1-256
NP_001161654.1/1-242
sp|P05408|7B2_HUMAN/1-212
XP_019625604.1/1-239
g13221.t1_Xenoturbella_bocki_7B2/1-225
tr|Q59E04|Q59E04_DROME/1-276
tr|V5TBY3|V5TBY3_PLADU/1-256
tr|A5GZV0|A5GZV0_APLCA/1-277
