# Supplementary material for: Insights into early animal evolution from the genome of the xenacoelomorph worm Xenoturbella bocki
Source: eLife. 2024 Aug 7;13:e94948. doi: 10.7554/eLife.94948 (PMC11521371; doi:10.7554/eLife.94948)
Supplement: Supplementary file 8. — X. bocki sequence is highlighted by a reddashed line. Sequences are available as Figure 8—source data 1; alignment is available at https://doi.org/10.5281/zenodo.6962271. [file elife-94948-supp8.pdf]

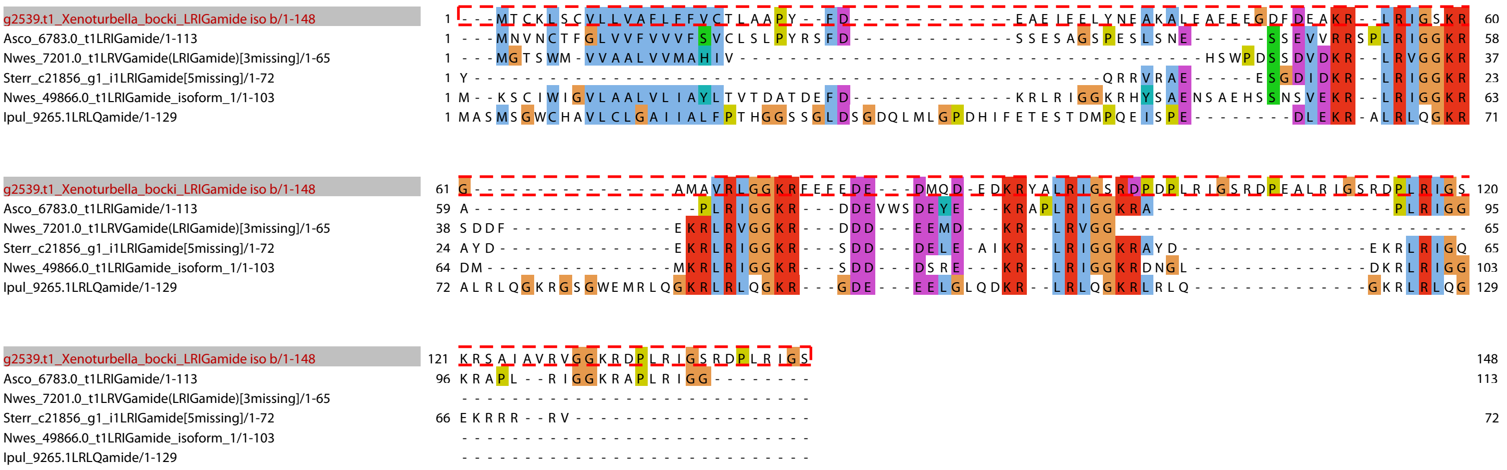

g2539.t1_Xenoturbella_bocki_LRIGamide iso b/1-148 1 ---MTCKLSCVLLVAFLFFVCTLAAPY--FD-------------EAEIFELYNEAKALEAEEEGDFDEAKR--LRIGSKR 60
Asco_6783.0_t1LRIGamide/1-113 1 ---MNVNCTFGLVVFVVVFSVCLSLPYRSFD-------------SSESAGSPESLSNE------SSEVVRRSPLRIGGKR 58
Nwes_7201.0_t1LRVGamide(LRIGamide)[3missing]/1-65 1 ---MGTSWM-VVAALVVMAHIV---------------------------------HSWPDSSDVDKR--LRVGGKR 37
Sterr_c21856_g1_i1LRIGamide[5missing]/1-72 1 Y---QRRVRAE-----ESGDIDKR--LRIGGKR 23
Nwes_49866.0_t1LRIGamide_isoform_1/1-103 1 M--KSCIWIGVLAALVLIAYLTVTDATDEFD-------------KRLRIGGKRHYSAENSAEHSSNSVEKR--LRIGGKR 63
Ipul_9265.1LRLQamide/1-129 1 MASMSGWCHAVLCLGAIIALFPTHGGSSGLDSGDQLMLGPDHIFETESTDMPQEISPE--------DLEKR-ALRLQGKR 71
g2539.t1_Xenoturbella_bocki_LRIGamide iso b/1-148 61 G---------------AMAVRLGGKRFEFEDE---DMQD-EDKRYALRIGSRDPDPLRIGSRDPEALRIGSRDPLRIGS 120
Asco_6783.0_t1LRIGamide/1-113 59 A------------------PLRIGGKR---DDEVWSDEYE---KRAPLRIGGKRA--------------------PLRIGG 95
Nwes_7201.0_t1LRVGamide(LRIGamide)[3missing]/1-65 38 SDDF--------------EKRLRVGGKR---DDD---EEMD---KR--LRVGG------------------------------ 65
Sterr_c21856_g1_i1LRIGamide[5missing]/1-72 24 AYD---------------EKRLRIGGKR---SDD---DELE-AIKR--LRIGGKRAYD----------------EKRLRIGQ 65
Nwes_49866.0_t1LRIGamide_isoform_1/1-103 64 DM----------------MKRLRIGGKR---SDD---DSRE---KR--LRIGGKRDNGL----------------DKRLRIGG 103
Ipul_9265.1LRLQamide/1-129 72 ALRLQGKRGSGWEMRLQGKRLRLQGKR---GDE---EELGLQDKR--LRLQGKRLRLQ----------------GKRLRLQG 129
g2539.t1_Xenoturbella_bocki_LRIGamide iso b/1-148 121 KRSAIAVRVGGKRDPLRIGSRDPLRIGS 148
Asco_6783.0_t1LRIGamide/1-113 96 KRAPL--RIGGKRAPLRIGG-------- 113
Nwes_7201.0_t1LRVGamide(LRIGamide)[3missing]/1-65 ----------------------------
Sterr_c21856_g1_i1LRIGamide[5missing]/1-72 66 EKRRR--RV------------------- 72
Nwes_49866.0_t1LRIGamide_isoform_1/1-103 ----------------------------
Ipul_9265.1LRLQamide/1-129 ----------------------------
