# Supplementary material for: Insights into early animal evolution from the genome of the xenacoelomorph worm Xenoturbella bocki
Source: eLife. 2024 Aug 7;13:e94948. doi: 10.7554/eLife.94948 (PMC11521371; doi:10.7554/eLife.94948)
Supplement: Supplementary file 9. — X. bocki sequence is highlighted by a reddashed line. Sequences are available as Figure 8—source data 1; alignment is available at https://doi.org/10.5281/zenodo.6962271. [file elife-94948-supp9.pdf]

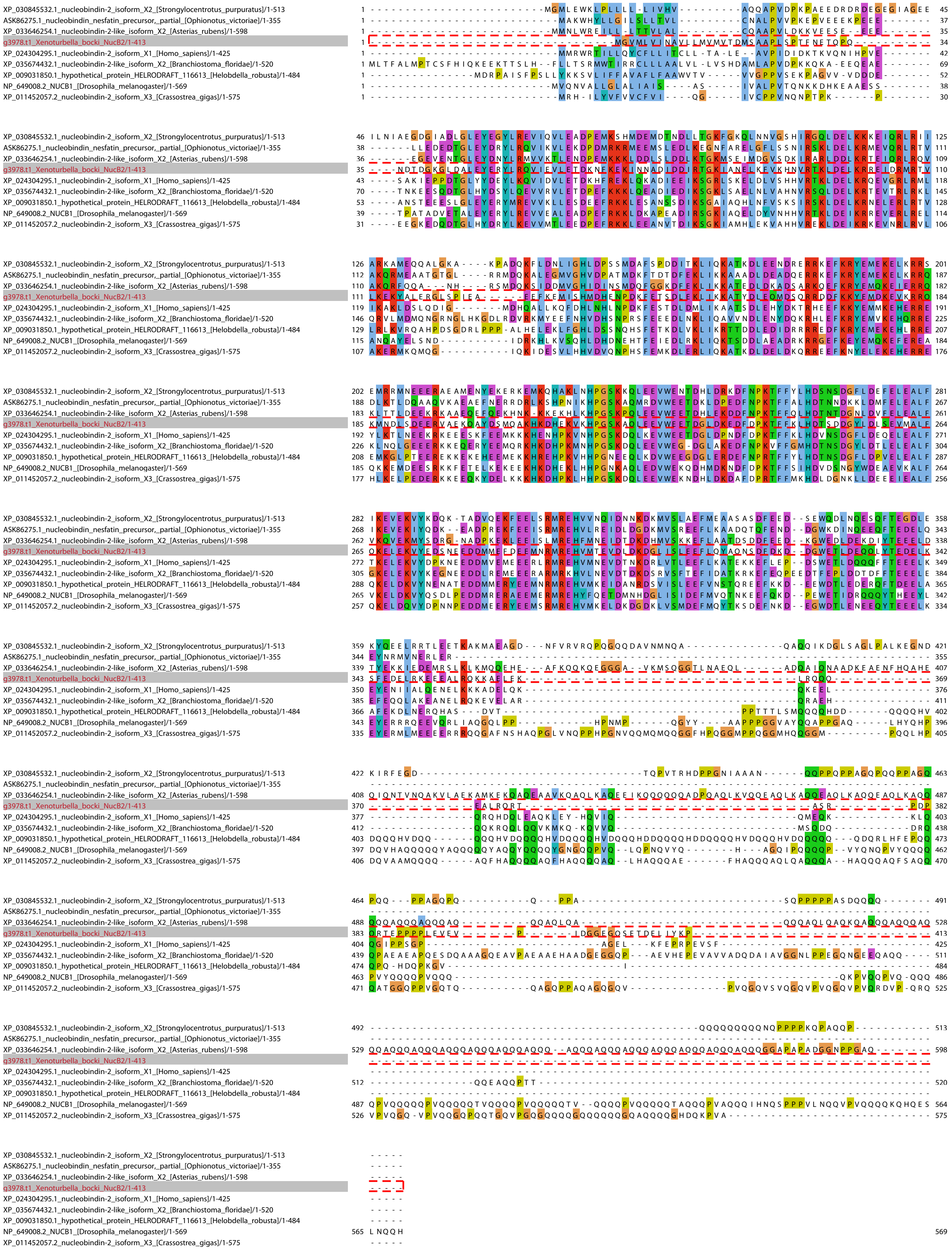
XP_030845532.1_nucleobindin-2_isoform_X2_[Strongylocentrotus_purpuratus]/1-513
ASK86275.1_nucleobindin_nesfatin_precursor,_partial_[Ophionotus_victoriae]/1-355
XP_033646254.1_nucleobindin-2-like_isoform_X2_[Asterias_rubens]/1-598
g3978.t1_Xenoturbella_bocki_NucB2/1-413
XP_024304295.1_nucleobindin-2_isoform_X1_[Homo_sapiens]/1-425
XP_035674432.1_nucleobindin-2-like_isoform_X2_[Branchiostoma_floridae]/1-520
XP_009031850.1_hypothetical_protein_HELRODRAFT_116613_[Helobdella_robusta]/1-484
NP_649008.2_NUCB1_[Drosophila_melanogaster]/1-569
XP_011452057.2_nucleobindin-2_isoform_X3_[Crassostrea_gigas]/1-575
