# Supplementary material for: Insights into early animal evolution from the genome of the xenacoelomorph worm Xenoturbella bocki
Source: eLife. 2024 Aug 7;13:e94948. doi: 10.7554/eLife.94948 (PMC11521371; doi:10.7554/eLife.94948)
Supplement: Supplementary file 10. — Red trianglehighlights the conserved cysteine positions. X. bocki sequence is highlighted by a red dashed line. Sequences are available as Figure 8—source data 1; alignment is available at https://doi.org/10.5281/zenodo.6962271. [file elife-94948-supp10.pdf]

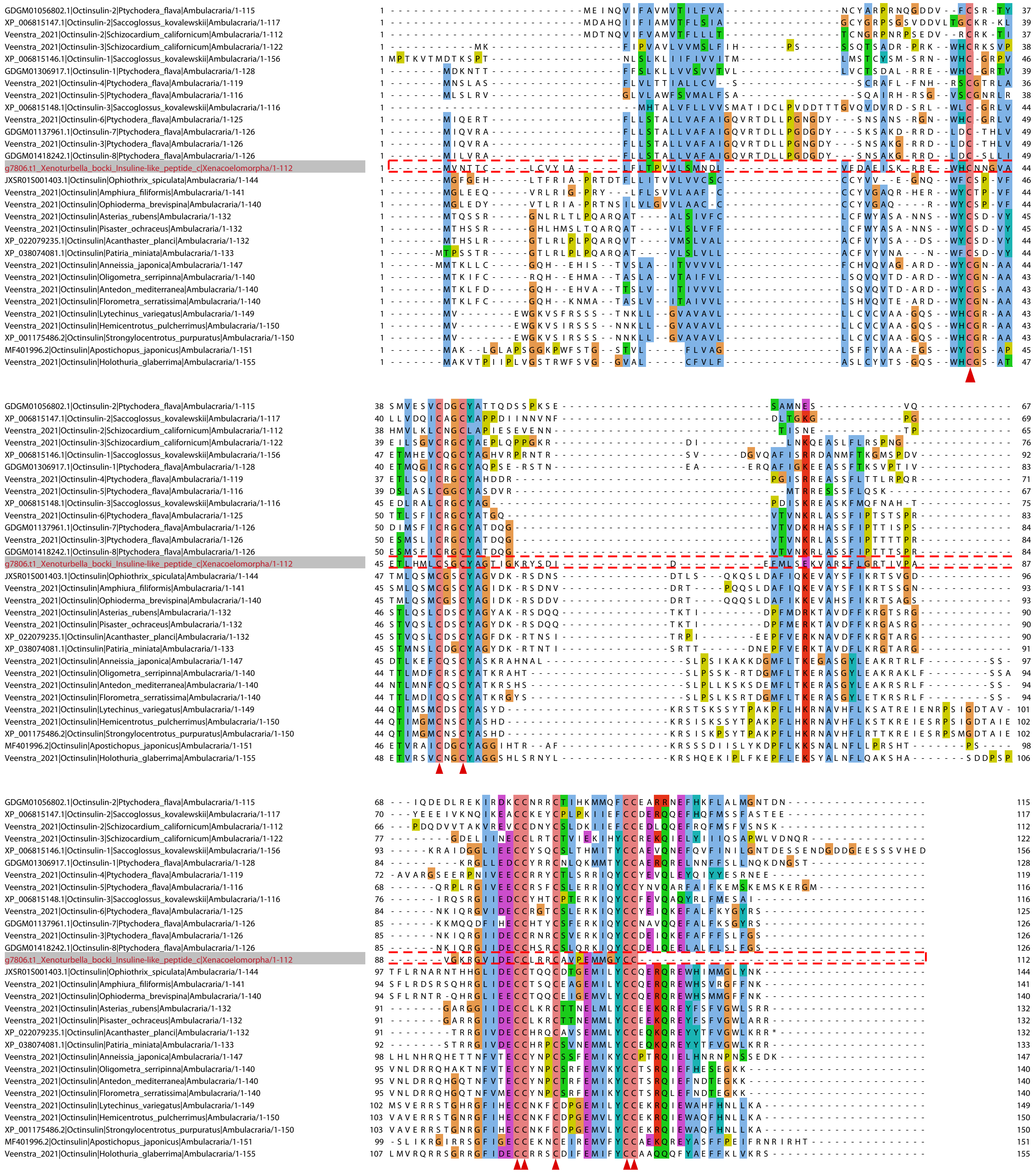
GDGM01056802.1|Octinsulin-2|Ptychodera_flava|Ambulacraria/1-115
XP_006815147.1|Octinsulin-2|Saccoglossus_kovalewskii|Ambulacraria/1-117
Veenstra_2021|Octinsulin-2|Schizocardium_californicum|Ambulacraria/1-112
Veenstra_2021|Octinsulin-3|Schizocardium_californicum|Ambulacraria/1-122
XP_006815146.1|Octinsulin-1|Saccoglossus_kovalewskii|Ambulacraria/1-156
GDGM01306917.1|Octinsulin-1|Ptychodera_flava|Ambulacraria/1-128
Veenstra_2021|Octinsulin-4|Ptychodera_flava|Ambulacraria/1-119
Veenstra_2021|Octinsulin-5|Ptychodera_flava|Ambulacraria/1-116
XP_006815148.1|Octinsulin-3|Saccoglossus_kovalewskii|Ambulacraria/1-116
Veenstra_2021|Octinsulin-6|Ptychodera_flava|Ambulacraria/1-125
GDGM01137961.1|Octinsulin-7|Ptychodera_flava|Ambulacraria/1-126
Veenstra_2021|Octinsulin-3|Ptychodera_flava|Ambulacraria/1-126
GDGM01418242.1|Octinsulin-8|Ptychodera_flava|Ambulacraria/1-126
g7806.t1_Xenoturbella_bocki_Insuline-like_peptide_c|Xenacoelomorpha/1-112
JXSR01S001403.1|Octinsulin|Ophiothrix_spiculata|Ambulacraria/1-144
Veenstra_2021|Octinsulin|Amphiura_filiformis|Ambulacraria/1-141
Veenstra_2021|Octinsulin|Ophioderma_brevispina|Ambulacraria/1-140
Veenstra_2021|Octinsulin|Asterias_rubens|Ambulacraria/1-132
Veenstra_2021|Octinsulin|Pisaster_ochraceus|Ambulacraria/1-132
XP_022079235.1|Octinsulin|Acanthaster_planci|Ambulacraria/1-132
XP_038074081.1|Octinsulin|Patiria_miniata|Ambulacraria/1-133
Veenstra_2021|Octinsulin|Anneissia_japonica|Ambulacraria/1-147
Veenstra_2021|Octinsulin|Oligometra_serripinna|Ambulacraria/1-140
Veenstra_2021|Octinsulin|Antedon_mediterranea|Ambulacraria/1-140
Veenstra_2021|Octinsulin|Florometra_serratissima|Ambulacraria/1-140
Veenstra_2021|Octinsulin|Lytechinus_variegatus|Ambulacraria/1-149
Veenstra_2021|Octinsulin|Hemicentrotus_pulcherrimus|Ambulacraria/1-150
XP_001175486.2|Octinsulin|Strongylocentrotus_purpuratus|Ambulacraria/1-150
MF401996.2|Octinsulin|Apostichopus_japonicus|Ambulacraria/1-151
Veenstra_2021|Octinsulin|Holothuria_glaberrima|Ambulacraria/1-155
